# sRNA Target Prediction Organizing Tool (SPOT) Integrates Computational and Experimental Data To Facilitate Functional Characterization of Bacterial Small RNAs

**DOI:** 10.1128/mSphere.00561-18

**Published:** 2019-01-30

**Authors:** Alisa M. King, Carin K. Vanderpool, Patrick H. Degnan

**Affiliations:** a Department of Microbiology, University of Illinois, Urbana, Illinois, USA; b Department of Microbiology and Plant Pathology, University of California, Riverside, Riverside, California, USA; University of Iowa

**Keywords:** Hfq, IntaRNA, RNA, RNase E, Starpicker, TargetRNA2

## Abstract

Small RNAs (sRNAs) posttranscriptionally regulate mRNA targets, typically under conditions of environmental stress. Although hundreds of sRNAs have been discovered in diverse bacterial genomes, most sRNAs remain uncharacterized, even in model organisms. Identification of mRNA targets directly regulated by sRNAs is rate-limiting for sRNA functional characterization. To address this, we developed a computational pipeline that we named SPOT for sRNA target prediction organizing tool. SPOT incorporates existing computational tools to search for sRNA binding sites, allows filtering based on experimental data, and organizes the results into a standardized report. SPOT sensitivity (number of correctly predicted targets/number of total known targets) was equal to or exceeded any individual method when used on 12 characterized sRNAs. Using SPOT, we generated a set of target predictions for the sRNA RydC, which was previously shown to positively regulate *cfa* mRNA, encoding cyclopropane fatty acid synthase. SPOT identified *cfa* along with additional putative mRNA targets, which we then tested experimentally. Our results demonstrated that in addition to *cfa* mRNA, RydC also regulates *trpE* and *pheA* mRNAs, which encode aromatic amino acid biosynthesis enzymes. Our results suggest that SPOT can facilitate elucidation of sRNA target regulons to expand our understanding of the many regulatory roles played by bacterial sRNAs.

**IMPORTANCE** Small RNAs (sRNAs) regulate gene expression in diverse bacteria by interacting with mRNAs to change their structure, stability, or translation. Hundreds of sRNAs have been identified in bacteria, but characterization of their regulatory functions is limited by difficulty with sensitive and accurate identification of mRNA targets. Thus, new robust methods of bacterial sRNA target identification are in demand. Here, we describe our small RNA target prediction organizing tool (SPOT), which streamlines the process of sRNA target prediction by providing a single pipeline that combines available computational prediction tools with customizable results filtering based on experimental data. SPOT allows the user to rapidly produce a prioritized list of predicted sRNA-target mRNA interactions that serves as a basis for further experimental characterization. This tool will facilitate elucidation of sRNA regulons in bacteria, allowing new discoveries regarding the roles of sRNAs in bacterial stress responses and metabolic regulation.

## Abstract (precis)

Small RNAs (sRNAs) regulate gene expression in diverse bacteria by interacting with mRNAs to change their structure, stability, or translation. Hundreds of sRNAs have been identified in bacteria, but characterization of their regulatory functions is limited by difficulty with sensitive and accurate identification of mRNA targets. Thus, new robust methods of bacterial sRNA target identification are in demand. Here, we describe our small RNA target prediction organizing tool (SPOT), which streamlines the process of sRNA target prediction by providing a single pipeline that combines available computational prediction tools with customizable results filtering based on experimental data. SPOT allows the user to rapidly produce a prioritized list of predicted sRNA-target mRNA interactions that serves as a basis for further experimental characterization. This tool will facilitate elucidation of sRNA regulons in bacteria, allowing new discoveries regarding the roles of sRNAs in bacterial stress responses and metabolic regulation.

## INTRODUCTION

Bacterial small RNAs (sRNAs) range in size from 50 to 530 nucleotides (nt). Regulation of mRNA targets by sRNAs via base pairing-dependent mechanisms alters translation or mRNA stability (1, 2). Most of the time, base pairing interactions involve the 5′ or 3′ untranslated region (UTR) of the target mRNA but can also involve sites within the coding region of the target mRNA. Small RNA-dependent translational repression often occurs via interactions that directly interfere with ribosome binding to the mRNA. However, sRNAs have also been shown to activate mRNA targets through various mechanisms, including interference with mRNA decay (3, 4). In recent years, it has become evident that sRNAs are ubiquitous and play an important role in mediating and regulating many basic cellular processes and stress responses. Hundreds of small RNAs have been identified in numerous bacterial species such as Bacillus subtilis (5), Listeria monocytogenes (6), and Salmonella enterica (7, 8). With the advancement of current technologies, the number of sRNAs identified in diverse organisms will surely increase. Consequently, there is a pressing need to develop new and better tools for sRNA characterization. In particular, there is a need for methods to address a major rate-limiting step in novel sRNA functional characterization, which is high-fidelity identification of mRNA targets.

A variety of computational and experimental methods have been used to predict and validate sRNA-mRNA target interactions. The computational tools currently available for sRNA target prediction, such as TargetRNA (9), sTarPicker (10), IntaRNA (11, 12), and CopraRNA (13), albeit powerful, have their limitations, the most problematic of which is the high rate of false-positive results. TargetRNA, sTarPicker, and IntaRNA all scan the entire genome and search for putative targets based on interaction hybridization energies. CopraRNA uses the same methodology as IntaRNA for predicting targets based on thermodynamic favorability of the interactions but goes a step further and also considers the conservation of those interactions across species, giving more weight to predictions that are conserved (13). When CopraRNA, IntaRNA, and TargetRNA were used in a side-by-side comparison, CopraRNA was found to have the highest positive predictive value (PPV) of 44% and reported the lowest rate of false-positive results for known sRNAs across 18 enterobacterial species (14). Although CopraRNA possesses the highest PPV out of all tools, there were still substantial false-positive results reported. Moreover, CopraRNA is limited to identifying conserved sRNA-target RNA interactions and cannot identify species-specific interactions. As a result, caution should be used with these individual algorithms, and they are frequently used in tandem with other target identification methods (14).

Experimental methods, including transcriptomic studies, have often been used to identify sRNA candidate targets. Transcriptomic methods uncover gene expression changes caused by the absence or overproduction of an sRNA. While microarrays and RNA sequencing have been successfully used to deduce sRNA targets, in many cases, separating direct effects from indirect effects is laborious and time-consuming. Moreover, the data obtained from transcriptomic studies can reveal only targets that are expressed under the specific growth conditions examined. As such, bona fide target genes that are poorly expressed or that are regulated by mechanisms that do not result in a substantial change in mRNA stability may be missed as sRNA targets. To address these issues, affinity purification methods have been developed to enhance identification of sRNA-mRNA interacting partners. For example, RIL-Seq (RNA interaction by ligation and sequencing) (15) identifies sRNA-mRNA partners that bind to the RNA chaperone Hfq (16) by coimmunoprecipitation, ligation, deep sequencing, and analysis of RNA chimeras, which often represent true interacting partners. MAPS (MS2 affinity purification coupled with RNA sequencing) (17) uses sRNA “bait” that is tagged with an MS2 aptamer and can be purified by interaction with the MS2 coat protein. RNA targets that are copurified with the sRNA bait are then identified by deep sequencing. Even with the variety of tools available for sRNA target identification, it is still not entirely clear which tools are the most effective for sRNA target identification. There has not been extensive validation of putative targets identified by affinity purification methods, but the authors of the RIL-seq study note that the sensitivity is on par with targets predicted by CopraRNA for well-characterized Escherichia coli sRNAs (15). The false-positive rate for affinity purification methods has not been quantified.

In order to streamline the use of multiple existing sRNA prediction algorithms, we developed a software pipeline called SPOT (sRNA target prediction organizing tool) that uses several algorithms in parallel to search for sRNA-mRNA interactions. The software collates predictions and allows integration of experimental data using customizable results filters. First, we used two well-characterized E. coli sRNAs, SgrS (18) and RyhB (19, 20), to assess the effectiveness of SPOT, as the targets of these sRNAs are well defined. Next, we extended the application of the SPOT pipeline to UTRs of mRNAs to identify potential sRNAs involved in regulation. We then applied the same parameters and analyses to a less-characterized E. coli sRNA, RydC. Employing a combinatorial approach through SPOT predictions and experimental validation, we were able to identify two new RydC targets, *pheA* and *trpE*, which were downregulated and upregulated, respectively, by RydC.

## RESULTS

### Integrated pipeline for sRNA target prediction algorithms.

**FIG 1:**
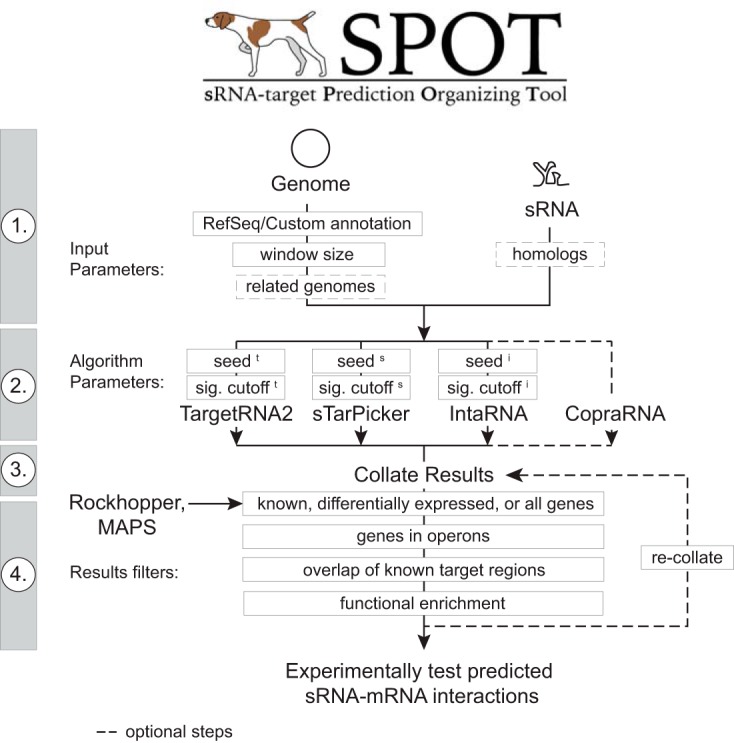
Schematic diagram of the SPOT pipeline. In step 1, a basic implementation of SPOT requires a user-provided reference genome and an sRNA sequence file. The user can customize the search window size and can optionally provide information required for CopraRNA (dashed boxes). In step 2, the user can set seed sizes and significance cutoffs for each algorithm (TargetRNA2 [superscript t], sTarPicker [superscript s], and IntaRNA [superscript i]). In step 3, SPOT runs the algorithms in parallel and generates a set of collated results. In step 4, the results filtering options shown narrow the list of predicted interactions to an experimentally tractable size for further validation or analysis.

A number of algorithms and tools for identifying putative sRNA-mRNA interactions have been developed (9, 10, 12, 13). However, no single target prediction tool is 100% accurate, the tools implement distinct user-defined parameters, each tool uses a different format for reporting results, and tools are hosted on distinct web platforms. Our approach was to create a single pipeline incorporating existing computational tools to search for sRNA binding sites, producing a collated and standardized result report (Fig. 1). We incorporated the TargetRNA2 (9), sTarPicker (10), IntaRNA (12), and CopraRNA (13) tools into this pipeline because they are widely used and have open source code. Input for the pipeline minimally includes a fasta sequence for the sRNA and the RefSeq number for the target genome. Additional RefSeq genome IDs and homologous sRNA sequences can be provided if the user wishes to include CopraRNA results in the analysis. The pipeline interface also allows the user to define a set of parameters for the individual algorithms and results filters. In particular, the results can be filtered for genes with known binding sites or sets of genes that were identified as putative targets by experimental methods (e.g., RNA-seq, MAPS [MS2 affinity purification coupled with RNA sequencing] [17], and RIL-seq [RNA interaction by ligation and sequencing] [15]). For instance, output from the RNA-seq analysis tool Rockhopper (21) can be used directly as a results filter. The program then follows four basic steps: (step 1) download/validate input files, (step 2) simultaneously initiate computational tools, (step 3) track job progress and read individual raw results, and (step 4) filter and collate results into a single report (Fig. 1). Finally, an option is provided that allows users to recollate the results from an initial analysis using different results filter settings.

### Pipeline optimization with SgrS and RyhB targets.

**FIG 2:**
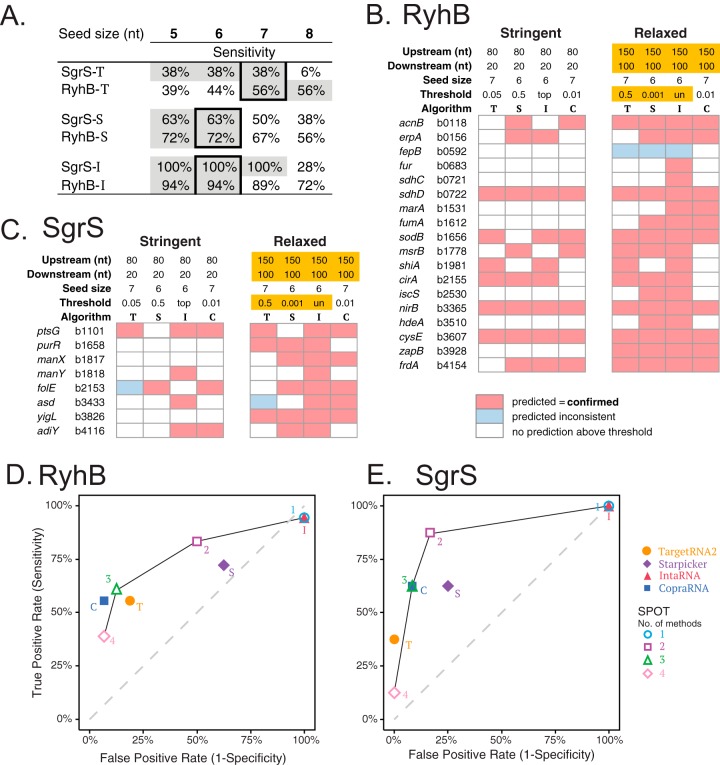
Validation of SPOT using known SgrS and RyhB sRNA-mRNA interactions. (A) Seed size indicates the number of consecutive base pairing nucleotides in an sRNA-mRNA interaction prediction. This is an adjustable parameter for each algorithm. Seed sizes were varied from 5 to 8 nt, and the sensitivity (true positive rate) was determined for known SgrS and RyhB interactions, while all other parameters were kept constant. Optimal seed sizes (shown in boxes outlined by thick black lines) were chosen for each algorithm. The highest percentage values for sensitivity are indicated with gray shading. Algorithms were abbreviated and are indicated after the sRNA or mRNA as follows: -T for TargetRNA2, -S for sTarPicker, and -I for IntaRNA. (B and C) Analyses were rerun using optimal seed sizes identified in panel A, but using a stringent parameter set with a narrow window size and high individual significance thresholds or a relaxed parameter set with a wider window size and low individual significance thresholds. Correctly predicted interactions for RyhB and SgrS are shown as pink cells, predictions that were inconsistent with confirmed interaction sites are shown in blue, and empty cells did not have any predictions above the indicated thresholds. Algorithms are abbreviated as follows: T for TargetRNA2, S for sTarPicker, I for IntaRNA, and C for CopraRNA. (D and E) RyhB and SgrS have experimentally validated (true-positive) and invalidated (true-negative) mRNA targets, which were used to generate receiver operating characteristic (ROC) curves. These plots enable assessment of the accuracy of SPOT and the individual algorithms. Using the relaxed search parameters, two-algorithm agreement in SPOT had greater true-positive rates and more acceptable false-positive rates compared with individual algorithms with the same settings.

SgrS and RyhB are two well-characterized model sRNAs in E. coli critical for glucose-phosphate (22) and iron limitation (23) stress responses, respectively. Numerous studies have confirmed 8 mRNA targets of SgrS (18, 24–27) and 18 of RyhB (19, 20, 28, 29). We used these two sRNAs to test the utility and sensitivity of the pipeline. For RyhB, the entire 90-nt sequence was used as the query for the bioinformatic search. For SgrS, only the 3′ 80 nt of the 227-nt sRNA was used as the query, since this is the region involved in target RNA binding. Our initial optimization of the pipeline focused primarily on three parameters: seed size, window size, and significance cutoffs. Each application utilizes distinct defaults for these parameters. For example, seed size, defined as the number of contiguous base pairing interactions required to define an sRNA-mRNA match is set at a default value of 7 in TargetRNA2 and IntaRNA and at 5 in sTarPicker. We varied the seed sizes for each algorithm and determined how different seed sizes impact the sensitivity of detection of true targets for SgrS and RyhB. Sensitivity is defined as the number of correctly predicted targets/number of total known targets (i.e., true positive rate). For TargetRNA2, a seed size of 7 gave the highest sensitivity for correct target predictions, with 38% and 56% correct predictions for SgrS and RyhB, respectively (Fig. 2A). For sTarPicker, the seed size giving the optimal sensitivity was 6, with 63% and 72% of known binding interactions identified for SgrS and RyhB, respectively. IntaRNA yielded the highest sensitivity of all three algorithms, again at a seed size of 6. IntaRNA correctly identified 100% of known SgrS interactions and 94% of known RyhB interactions (Fig. 2A). On the basis of these results, we used seed size settings of 7 for TargetRNA2 and 6 for IntaRNA and sTarPicker for all other analyses.

Next, we evaluated how altering the window size and significance cutoffs impacted the accuracy of predictions (Fig. 2B and C). The window size refers to the size of the region upstream and downstream of every start codon in the genome that is searched for potential base pairing with the query sRNA. The default window sizes for the tools vary dramatically. The default TargetRNA2 window size is 80 nt upstream and 20 nt downstream (80/20) of each start codon (9). The default for sTarPicker is 150/100, the default for IntaRNA is 75/75, and the default for CopraRNA is 200/100. Likewise, the tools have different metrics to determine the significance of a match either providing a *P* value (TargetRNA2, IntaRNA, and CopraRNA) or a probability measure (sTarPicker). TargetRNA2 generates *P* values for predicted interactions based on the sRNA-mRNA hybridization energy scores of a randomized mRNA pool (9). IntaRNA utilizes *P* values based on transformation of the energy scores calculated for all putative target binding sites with energy score of ≤0 (11, 12). CopraRNA combines individual IntaRNA *P* value predictions among clusters of genes to generate a weighted *P* value and false discovery rate (FDR)-corrected q-value (13). In contrast, sTarPicker uses a machine learning approach to generate probabilities as a proportion of base classifiers (*n* = 1,000) that support each proposed interaction (10). The sTarPicker authors report that probabilities of ≥0.5 correspond to likely sRNA-mRNA interactions. SPOT provides the user with the ability to alter the search window and significance thresholds used by all the algorithms included in the pipeline (Fig. 1). We chose two sets of parameters that we define as stringent and relaxed and tested the performance of each set of parameters in correctly identifying known RyhB and SgrS target binding sites (Fig. 2B and C). Stringent parameters incorporated a window size of 80 nt upstream and 20 nt downstream (80/20) of start codons as the search region and significance thresholds of 0.05 for TargetRNA2, 0.5 for sTarPicker, “top” (e.g., the top 100 predictions) for IntaRNA, and 0.01 for CopraRNA (Fig. 2B and C). Relaxed parameters used a comparatively larger window size of 150/100 and thresholds of 0.5, 0.001, “un,” (e.g., all predictions) and 0.01 for TargetRNA2, sTarPicker, IntaRNA, and CopraRNA, respectively.

Using stringent search parameters, 10/18 known RyhB target binding sites and 2/8 known SgrS target binding sites were correctly predicted by ≥2 algorithms (Fig. 2B and C, indicated by 2 or more pink cells and absence of blue cells). Using relaxed parameters, the correctly predicted interactions rose to 17/18 and 6/8 for RyhB and SgrS, respectively. Thus, for both RyhB and SgrS, relaxed parameters substantially increased the number of correctly identified binding sites (Fig. 2B and C). Notably, the use of relaxed parameters was necessary to capture true binding sites like the SgrS binding site on *yigL* mRNA, which is located further from the start codon than is typical. The relaxed parameters improve the sensitivity of individual methods but may result in the downside of identifying more false-positive results. IntaRNA has a high sensitivity for true-positive results (correct identification of known sRNA binding sites) under the relaxed settings but also gives a high rate of likely false-positive results, illustrated by the fact that IntaRNA predicts >3,400 binding interactions that are not predicted by any other algorithm. Mitigating this downside of using relaxed parameters, we saw that in the majority of instances, the correct RyhB and SgrS binding sites were predicted by ≥2 methods, and incorrect predictions by ≥2 methods occurred rarely (1/18 for RyhB and 0/8 for SgrS) (Fig. 2B and C).

For SgrS and RyhB, at least a dozen mRNAs have been experimentally defined as nontargets for each sRNA (18). In other words, predicted sRNA-mRNA interactions were tested and shown not to mediate regulation of the mRNA in question. These examples served as controls that allowed us to calculate false-positive rates. Together with the sensitivity measures for each algorithm and the pipeline, we generated receiver operating characteristic (ROC) curves to assess the accuracy of the methods alone and in combination (Fig. 2D and E). Ideally, tools should yield high true-positive rates and low false-positive rates, resulting in values falling in the upper left quadrant of the ROC curve. Our results indicate that when two methods converge on the same prediction, the pipeline achieves ≥75% sensitivity and ≤50% false-positive rate for both sRNAs. This is a marked improvement in most instances over the single algorithms used here (Fig. 2D and E). In particular, using a two-method threshold mitigates the very high false-positive rate of IntaRNA. We note that making the IntaRNA *P* value cutoff more stringent (e.g., 0.05) decreases the false-positive rate dramatically but at a cost to sensitivity (see Fig. S1 in the supplemental material). Similarly, requiring three or four algorithms to identify the same predicted interaction decreases the false-positive rates of predictions for RyhB and SgrS; however, the sensitivity decreases by more than 25% (Fig. 2D and E). Collectively, these analyses suggest that the use of relaxed search parameters and a combined evidence approach requiring a minimum of two algorithms to predict the same binding interaction is an effective means of improving sRNA target prediction sensitivity.

10.1128/mSphere.00561-18.2 FIG S1 SPOT 2 method agreement is more effective than adjustment of IntaRNA *P* value cutoff. The true- and false-positive rates for SPOT using relaxed parameters with requirement for ≥2 identical binding site predictions (purple open squares labeled 2) or for IntaRNA with no *P* value cutoff (red triangles labeled un for no cutoff), or *P* value cutoffs of 0.05 ( red circles labeled 0.05) or 0.01 (red squares labeled 0.01) for RyhB (A) and SgrS (B). The use of SPOT with ≥2 identical binding site predictions maintains the highest sensitivity (true-positive rate) with a moderate false-positive rate. Download FIG S1, PDF file, 0.2 MB. Copyright © 2019 King et al. 2019 King et al. This content is distributed under the terms of the Creative Commons Attribution 4.0 International license.

The SPOT pipeline accepts several results filters to facilitate analysis of the predictions. First, users can provide the program a list of binding site locations for known mRNA targets (e.g., true-positive results). Second, users can include genes on the list that lack known binding sites in order to limit the results reporting to select genes of interest, for example those that emerged from experimental analyses (e.g., RNA-seq). Integration of experimental data with computational predictions is another valuable way of reducing potential false-positive predictions.

On the basis of our results and observations during the optimization of SgrS and RyhB target identification, we designed SPOT to prioritize the target binding site predictions (Fig. S2). First, known binding sites correctly predicted by ≥2 algorithms (rank 1) or 1 algorithm (rank 2) are reported. Any gene targets with predictions that are discordant with known binding sites (rank 3) are reported next. Then any additional targets with the same predicted target site found by ≥2 algorithms are ranked next (rank 4). This is followed by targets that were predicted by only a single algorithm, in the following order: CopraRNA (rank 5), TargetRNA or sTarPicker (rank 6), and IntaRNA (rank 7). Using the results filters, a user can narrow or widen their searches, for example, by limiting the predictions made by single algorithms or by applying secondary filters on binding site regions.

10.1128/mSphere.00561-18.3 FIG S2 SPOT produces ranked predictions of sRNA-mRNA interactions. SPOT was used to analyze SgrS using relaxed parameters and a gene list that contained known binding sites as well as genes known to be differentially regulated during SgrS pulsed overexpression (18). (A) Resulting predictions are ranked based on known binding sites correctly predicted by ≥2 algorithms (rank 1), known binding sites correctly predicted by 1 algorithm (rank 2), predictions that are inconsistent with known binding sites (rank 3), and additional targets with ≥2 identical algorithm predictions (rank 4). The remaining targets were predicted by only a single algorithm as follows: CopraRNA (rank 5), TargetRNA or Starpicker(rank 6), or ) IntaRNA (rank 7. (B) Excel and tab separated files generated by SPOT include vital statistics regarding the putative interactions and include the alignment plots generated by the individual algorithms. Download FIG S2, PDF file, 0.2 MB. Copyright © 2019 King et al. 2019 King et al. This content is distributed under the terms of the Creative Commons Attribution 4.0 International license.

### Application of SPOT to additional sRNAs.

**FIG 3:**
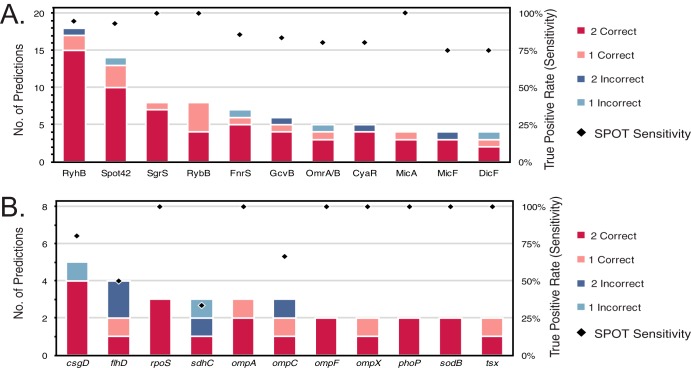
SPOT demonstrates high sensitivity for detecting targets of multiple sRNAs. (A) Along with RyhB and SgrS, nine additional sRNAs were analyzed with SPOT using the relaxed parameter set, demonstrating the robustness of the SPOT pipeline for correctly identifying sRNA-mRNA target interactions. Stacked bars show the number of experimentally validated mRNA targets correctly or incorrectly identified by 1 or ≥2 methods. Black diamonds indicate the overall true-positive rate (sensitivity) of SPOT for each sRNA. (B) Eleven UTRs that are experimentally validated to interact with multiple sRNAs were used in a “reverse” search in SPOT (i.e., using the UTR as the query and the sRNAs as the targets). The average sensitivity of this method is lower than in panel A; however, this is a novel means for identifying sRNAs that might affect genes of interest. Plots are drawn as in panel A.

To evaluate the robustness of the defined pipeline parameters and our ranking methods, we ran similar analyses on 9 additional sRNAs with ≥4 known targets. Overall, we found that the SPOT pipeline sensitivity (e.g., the percentage of correctly identified interactions) was equal to or exceeded any individual method (average of 84% ± 8.5%; Fig. 3A and Fig. S3A). As before, we found that correct identification by ≥2 methods occurred in the majority of instances (Fig. 3A, red bars). The full list of target predictions generated by ≥2 methods for all 11 sRNAs (Fig. S3B) are included as Data Set S1. On average, the primary analysis by the pipeline took 1 h 15 min ± 35 min, using as many as six processing cores simultaneously. Recollation of the results using different filters took only an average of 29 s ± 6s.

10.1128/mSphere.00561-18.4 FIG S3 SPOT successfully identified known sRNA-mRNA interactions and finds potential novel interactions by consensus. (A) True-positive rates for individual algorithms were compared among 11 sRNAs analyzed with relaxed parameter searches. In nearly all cases, SPOT shows greater sensitivity than CopraRNA, Starpicker, and TargetRNA2. SPOT and IntaRNA show identical sensitivities, but IntaRNA alone yields a very high false-positive rate (Fig. S1). (B) SPOT was run with relaxed parameters without filtering on lists of known targets. All predictions are shown for each sRNA, and stacked bars indicate the percentage of predictions that were shared by one, two, three, or four algorithms. The bulk of single-algorithm predictions were made by IntaRNA, and the majority of these are likely false-positive results. Using a two-algorithm agreement cutoff yields between 300 and 900 predictions per sRNA (Data Set S1). Download FIG S3, PDF file, 0.4 MB. Copyright © 2019 King et al. 2019 King et al. This content is distributed under the terms of the Creative Commons Attribution 4.0 International license.

10.1128/mSphere.00561-18.9 DATA SET S1 SPOT output for 12 E. coli small RNAs. Download Data Set S1, XLSX file, 1.9 MB. Copyright © 2019 King et al. 2019 King et al. This content is distributed under the terms of the Creative Commons Attribution 4.0 International license.

### Extended application of the SPOT pipeline with mRNA as the query sequence.

The four individual algorithms are intended to identify the interaction of an sRNA with mRNA targets. However, a user may be interested in determining which known sRNAs interact with a specific mRNA of interest. Normally this would require running an individual search for each of the tens to hundreds of sRNAs from that organism. As part of our pipeline we have designed a feature that allows a user to input a custom annotation file for their reference genome. Therefore, instead of providing the list of mRNA targets, sRNAs can be provided to the algorithm and the relevant mRNA sequence, e.g., a 5′ untranslated region (UTR) of interest can be used as the query. We carried out this “reverse” analysis on 11 E. coli 5′ UTRs that have already been demonstrated to interact with ≥2 different sRNAs. The results are comparable to the analysis using sRNAs as targets—known sRNA interactions were identified with an average sensitivity of 85% ± 24% (Fig. 3B). Moreover, using the two-algorithm cutoff, we were able to use this approach to predict 5 to 14 additional sRNAs that putatively bind the UTRs and could affect their regulation (Data Set S1). We note that due to technical constraints, the reverse search method can be used only with TargetRNA2, sTarPicker, and IntaRNA at this time. This approach is a novel feature that will facilitate ongoing sRNA research.

### Examination of novel RydC target predictions.

We next sought to use the SPOT pipeline to identify additional targets for the poorly characterized sRNA RydC. RydC was reported to repress *yejA* mRNA (encoding an uncharacterized ABC transporter [30]) and *csgD* mRNA (encoding the master regulator of curli biogenesis [31]), but the molecular mechanisms of RydC-mediated repression were not reported. Fröhlich et al. (3) demonstrated that RydC activates *cfa* mRNA, encoding cyclopropane fatty acid synthase. This activation involves RydC-dependent protection of *cfa* mRNA from RNase E-mediated degradation (3). Despite identification of these targets, the physiological function of RydC remains unclear. We used SPOT to identify additional targets of RydC as a means to gain further insight into its physiological role in E. coli.

Our strategy for RydC target identification was to combine computational and experimental data to generate an experimentally tractable list of putative targets for further validation. Experimental identification of putative targets was accomplished by pulse expression of RydC from an inducible promoter followed by identification of RydC-dependent changes in gene expression by RNA-seq. Vector control and P*_lac_*-*rydC* plasmids were maintained in a Δ*rydC* host strain grown in rich medium (LB) at 37°C. Expression of *rydC* was induced by the addition of IPTG to cultures, and total RNA was harvested at 10 min after induction. RNA-seq data output fastq files were analyzed with Rockhopper and exported as .xls files (Data Set S2). A total of 158 genes met our criteria for differential expression (see Materials and Methods) in RydC-expressing cells compared to control cells (Data Set S2).

10.1128/mSphere.00561-18.10 DATA SET S2 RNA-seq differential expression in RydC pulse expression and control strains. Download Data Set S2, XLSX file, 0.4 MB. Copyright © 2019 King et al. 2019 King et al. This content is distributed under the terms of the Creative Commons Attribution 4.0 International license.

**FIG 4:**
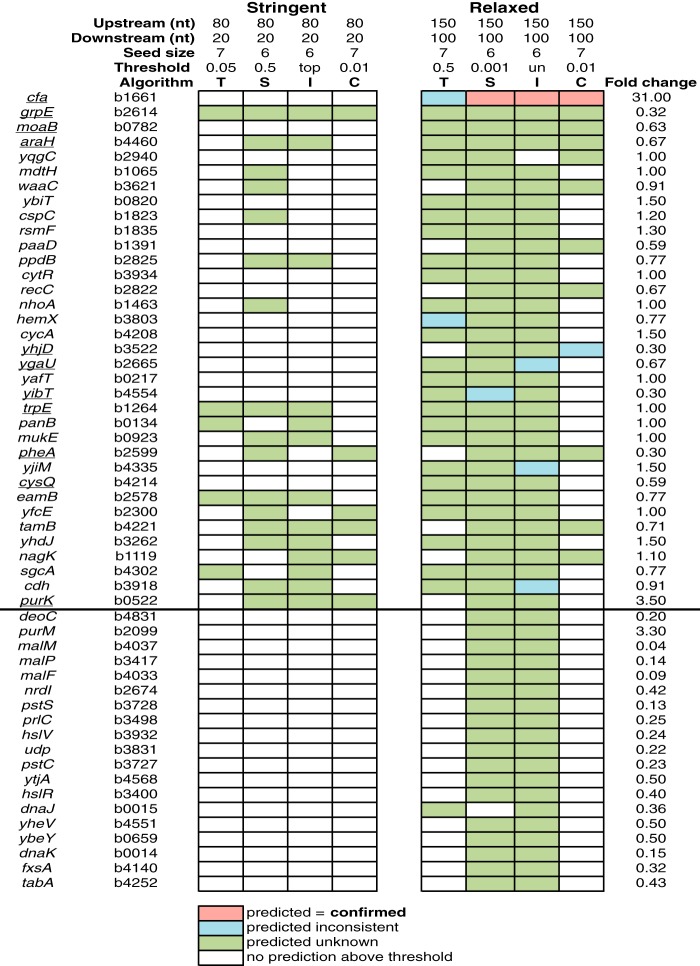
SPOT predictions for the sRNA RydC. Analyses were run with optimal seed sizes as determined in Fig. 2. Genes above the thick black line denote those with ≥3 computational predictions, while genes below the thick black line had 2 computational predictions and differential RNA-seq expression (fold change of ≥ 1.5 or ≤0.5, q-value of ≤0.005). Correctly predicted interactions for RydC are shown as pink cells, unknown predictions that were consistent among algorithms are shown in green, inconsistent predictions are shown in blue, and empty cells did not have any predictions above the indicated thresholds. Algorithms are abbreviated as follows: T for TargetRNA2, S for sTarPicker, I for IntaRNA, and C for CopraRNA. Underlined genes were chosen for experimental validation (Table 1).

To identify putative RydC targets, the SPOT pipeline was applied to RydC using both stringent and relaxed parameters, with the former being more restrictive for window size and algorithm thresholds as described above (see the RydC tab in Data Set S1 for the full Excel table output). Similar to analyses for SgrS and RyhB, the relaxed parameters yielded a greater number of predictions than the stringent parameters. A subset of the SPOT output is shown in Fig. 4, with potential targets that were predicted by ≥3 algorithms with the relaxed parameters shown above the thick black line. The RydC binding site for a validated target, *cfa* mRNA, was correctly predicted by three algorithms in the relaxed run. TargetRNA2 predicted a binding site that was inconsistent with the known binding site. The *cfa* prediction was absent in the stringent run, since the base pairing interaction between RydC and *cfa* mRNA takes place outside the window specified in the stringent run (Fig. 4). Some of the putative targets predicted by ≥3 algorithms were also differentially expressed in RydC pulse expression RNA-seq experiments (indicated in the Fold change column in Fig. 4). Another set of genes were predicted as targets by ≥2 algorithms and differentially expressed in RNA-seq experiments (Fig. 4, see targets below the thick black line).

Genes chosen for further analysis are listed in Table 1, along with information about their functions, differential expression in RNA-seq, predicted binding interactions, and algorithm predictions. Genes underlined in Fig. 4 were chosen for further study (and included in Table 1) if they had ≥3 identical predictions from base pairing algorithms, and/or met the RNA-seq cutoff for differential expression in RydC-expressing cells compared to control cells. Several other genes that did not meet the criteria for inclusion in Fig. 4 were also chosen for analysis because they had been described previously as RydC targets or because they encode proteins belonging to functional categories related to known RydC targets (Table 1).

**TABLE 1:** List of putative RydC targets chosen for further testing

Gene	Putative function	Fold change (P*_lac_*-*rydC*/vector)^*a*^	Predicted interactions^*b*^	Algorithm predictions^*c*^	Reference
*cfa*	Cyclopropane fatty acyl phospholipid synthase	31.00	*cfa* −110 to −98	T, S, I, C	3
			RydC +14 to +2		

*grpE*	Nucleotide exchange factor	0.32	*grpE* +16 to +29	T, S, I, C	This study
			RydC +64 to +51		

*moaB*	Part of *moaABCDE* operon	0.63	*moaB* +27 to +42	T, S, I, C	This study
			RydC +44 to +29		

*araH*	Arabinose ABC transporter membrane subunit	0.67	*araH* +9 to +25	T, S, I, C	This study
			RydC +17 to +1		

*yhjD*	Putative transporter	0.30	*yhjD* −62 to −11	S, I, C	This study
			RydC +53 to +2		

*ygaU*	Potassium binding protein (Kbp)	0.67	*ygaU* +78 to +94	T, S, I	This study
			RydC +31 to +15		

*yibT*	Protein YibT	0.30	*yibT* −24 to −10	T, S, I	This study
			RydC +28 to +14		

*trpE*	Anthranilate synthase subunit	1.00	*trpE* +12 to +22	T, S, I	This study
			RydC +47 to +37		

*pheA*	Fused chorismate mutase/prephenate dehydratase	0.30	*pheA* +4 to +11	S, I, C	This study
			RydC +10 to +3		

*cysQ*	3′(2′),5′-Bisphosphate nucleotidase	0.59	*cysQ* +48 to +67	T, S, I	This study
			RydC +21 to +2		

*purK*	5-(Carboxyamino) imidazole ribonucleotide synthase	3.49	*purK* −38 to −19	S, I, C	This study
			RydC +59 to +5		


*csgD*	DNA binding transcriptional dual regulator	0.75	*csgD* −19 to +3	I	31
			RydC +26 to +5		

*yejA*	Putative oligopeptide ABC transporter periplasmic component	0.91	*yejA* +1265 to +1273	I	30
			RydC +47 to +39		

*lldR*^*d*^	DNA-binding transcriptional dual regulator	1.17	*lldR* +15 to +65	S	This study
			RydC +61 to +15		

a The ratios determined from RNA-Seq experiments (see Data Set S1 in the supplemental material).

b The bases involved in the interaction in the 5′-to-3′ direction for the target and in the 3′-to-5′ direction for RydC in relation to the +1 site (start of translation).

c The algorithms that predicted a base pairing interaction are indicated as follows: T, TargetRNA2; S, sTarPicker; I, IntaRNA; C, CopraRNA.

d *lldR* was chosen for further analysis based on observed regulation by a putative RydC regulator (C. M. Bianco and C. K. Vanderpool, unpublished data).

### Testing pipeline predictions for RydC.

**FIG 5:**
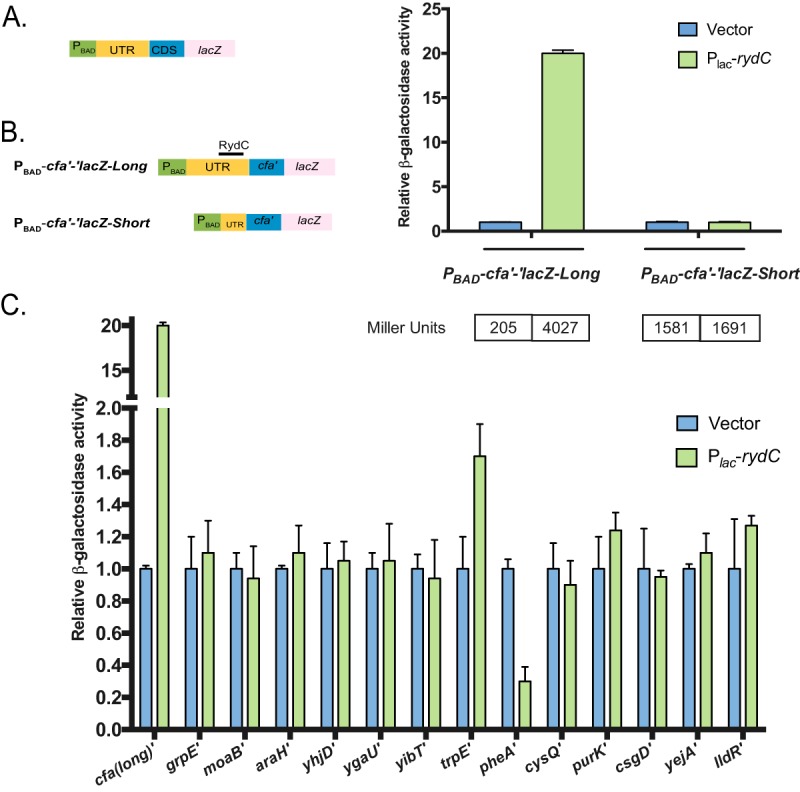
Validation of RydC target predictions. (A) The design for the translational *lacZ* constructs is shown. The arabinose promoter (P_BAD_) (green box), untranslated region (UTR) (yellow box), coding sequence (CDS) (blue box), and *lacZ* gene (pink box) are indicated. (B, left) To confirm *cfa* as a RydC target, both full-length and shortened *cfa′-′lacZ* translational fusions were tested in backgrounds with vector or P_lac_-*rydC* plasmids. Expression of the reporter fusion was induced with 0.002% L-arabinose, while induction of RydC was achieved with 0.1 mM IPTG. (Right) The activities were normalized to vector control and plotted as relative activity. These experiments were conducted as three independent trials with three biological replicates per trial. The specific activity values (in Miller units) are shown below the graph. Error bars represent standard deviations among biological replicates from a representative trial. (C) Empty vector or RydC was overexpressed in strains with reporter fusion as indicated above. Expression of the fusion and RydC was induced as previously described. As a comparison, the positive-control *cfa(long)′* was included in the experiment. These experiments were conducted as three independent trials with three biological replicates per trial. Error bars represent standard deviations among biological replicates from a representative trial. Fold differences in other trials were consistent with the trial shown.

To test the targets selected for further validation for regulation by RydC, we constructed translational fusions to putative targets. These fusions were placed under the control of an arabinose-inducible promoter (P_BAD_) to eliminate possible indirect transcriptional effects. For each target, the entire 5′ UTR and part of the coding sequence (length variable, depending on the location of the predicted RydC binding site) was fused to *'lacZ* (Fig. 5A). Strains containing the reporter fusions were transformed with vector control or P*_lac_*-*rydC* plasmids, and reporter activity was measured after induction with IPTG.

In *Salmonella enterica,* RydC was demonstrated to activate *cfa* translation by occluding an RNase E cleavage site to stabilize the *cfa* mRNA (3). Conservation of RydC-*cfa* mRNA interactions between E. coli and *Salmonella* and SPOT identification of *cfa* as a putative RydC target (Fig. 4 and Table 1) suggest that E. coli RydC regulates *cfa* in a similar manner. To confirm this, we constructed two translational fusions: P_BAD_-*cfa*'-'*lacZ*-Long, which contains the RydC binding site, and P_BAD_-*cfa*'-'*lacZ*-Short, which lacks the RydC binding site (Fig. 5B). RydC production strongly activated the long fusion, increasing activity by >20-fold compared to the vector control strain (Fig. 5B). As expected, activity of the short fusion lacking the RydC binding site was unaffected upon RydC induction (Fig. 5B). These results support the model that *cfa* mRNA is directly regulated by RydC in both S. enterica and E. coli.

Strains harboring reporter fusions to 13 other putative targets (listed in Table 1) were transformed with vector control and P*_lac_-rydC* plasmids, and β-galactosidase assays were performed after a period of RydC induction (Fig. 5C). Only two of the target fusions were differentially regulated by the criteria we selected (≥1.5-fold or ≤0.5-fold) in cells expressing RydC compared to the vector control (Fig. 5C). These two targets were *pheA* and *trpE*, which both encode proteins involved in aromatic amino acid biosynthesis. Previous studies (30, 31) reported RydC-dependent translational repression of the *yejA* and *csgD* mRNAs, though we note that specific and direct base pairing interactions with RydC were not demonstrated. Our translational fusions to these putative targets did not show any differential regulation in response to RydC expression (Fig. 5C).

### RydC regulates genes in aromatic amino acid biosynthetic pathways.

**FIG 6:**
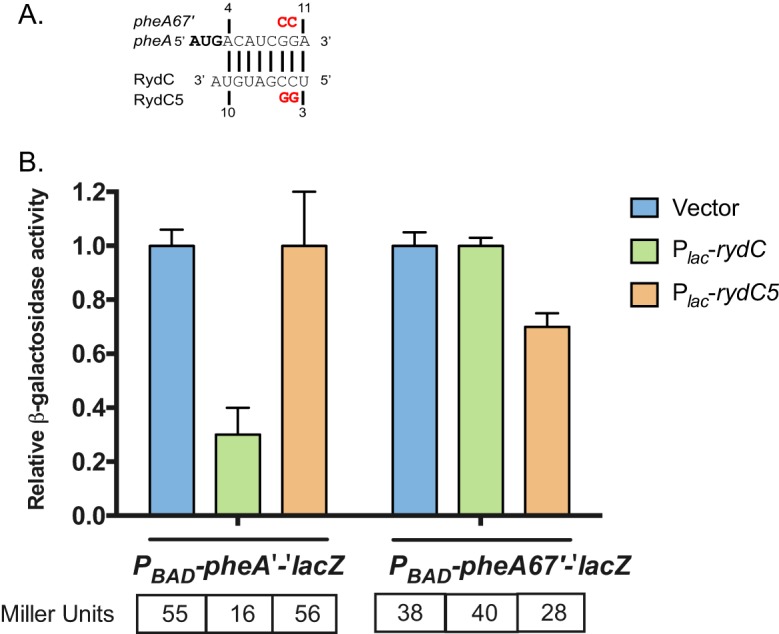
RydC represses *pheA* translation. (A) The predicted base pairing between *pheA* mRNA and RydC from IntaRNA. The residues highlighted in red represent point mutations made for each of the variant fusions/RydC alleles. The numbers are in relation to the +1 of RydC and the AUG of *pheA*. To test *pheA* as a putative target, both full-length and mutated (*pheA67*′) *pheA*′*-*′*lacZ* translational fusions were tested. (B) RydC or a RydC variant (RydC5) was overexpressed in the *pheA*′ and *pheA67*′*-*′*lacZ* fusion backgrounds. Expression of the fusion and RydC was induced as described in the legend to Fig. 5. The activities were normalized to vector control and plotted as relative activity. The data were analyzed and reported as described in the legend to Fig. 5. The specific activity values (in Miller units) are presented below the graph.

In RNA-Seq experiments, the levels of *pheA* mRNA were reduced to ∼30% of control levels when RydC was ectopically expressed (Data Set S2). Likewise, in RydC-producing cells, activity of the P_BAD_-*pheA'-'lacZ* fusion was ∼30% that of the vector control (Fig. 5C). The predicted RydC-*pheA* mRNA base pairing interaction involves the 5′ end of RydC and the coding region of *pheA,* directly adjacent to the start codon (Fig. 6A). The P_BAD_-*pheA*'-'*lacZ* fusion encompasses all of the 5′ UTR and 645 nt of the coding region. A reporter derived from this has mutations that disrupt the predicted base pairing with RydC, resulting in the P_BAD_-*pheA67*'-'*lacZ* fusion with mutations G9C/G10C (Fig. 6A). A *rydC* allele with compensatory mutations (C4G/C5G) was constructed and named RydC5. The mutations in RydC5 abrogated regulation of the wild-type P_BAD_-*pheA*'-'*lacZ* fusion. Likewise, the mutations in P_BAD_-*pheA67*'-'*lacZ* prevented regulation by wild-type RydC. The compensatory mutant pair P_BAD_-*pheA67*'-'*lacZ* and RydC5 had restored regulation, albeit not to fully wild-type levels. Together, these data suggest that RydC targets *pheA* mRNA for translational repression. Due to the location of the base pairing interaction in the translation initiation region, the mechanism is likely direct occlusion of ribosome binding to *pheA* mRNA by RydC.

**FIG 7:**
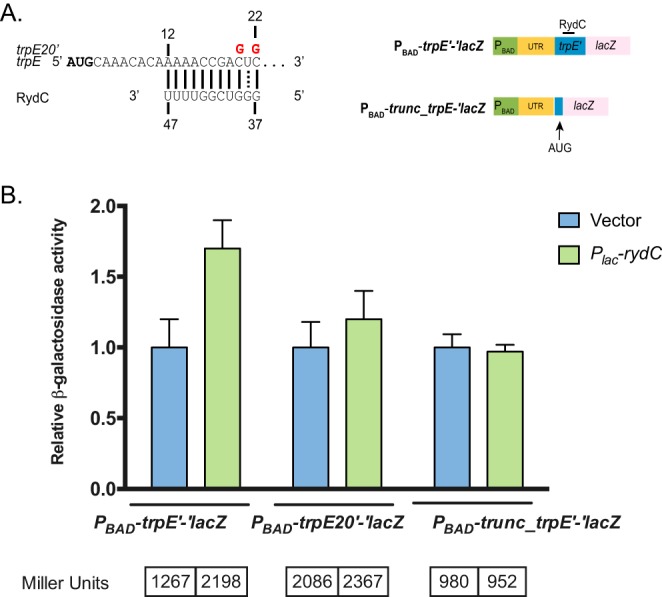
RydC activates *trpE* translation. (A) The predicted base pairing between *trpE* mRNA and RydC from IntaRNA. The vertical/dotted lines represent the seed region for base pairing interactions. The residues highlighted in red represent point mutations made for each of the variant fusions/RydC alleles. The numbers are in relation to the +1 site of RydC and the AUG of *trpE*. To test *trpE* as a putative target, both full-length, mutated (*trpE20*′), and truncated (*trunc_trpE*′) *trpE*′*-′lacZ* translational fusions were tested. (B) Empty vector or RydC was overexpressed in the *trpE*′, *trpE20′*, and *trunc_trpE*′-′*lacZ* fusion backgrounds. Expression of the fusion and/or RydC was induced as previously described. The experiments were conducted and data were analyzed and presented as described in the legend to Fig. 5. The specific activity values (in Miller units) are presented below the graph.

Another new putative RydC target is *trpE,* which encodes a component of the anthranilate synthase involved in tryptophan biosynthesis. A P_BAD_-*trpE*'-'*lacZ* fusion encompassing the 30-nt *trpE* mRNA 5′ UTR and 42 nt of *trpE* coding sequence was activated upon RydC production by slightly less than twofold (Fig. 5C and 7B). The predicted RydC-*trpE* mRNA base pairing interaction involves sequences near the 3′ end of RydC and sequences within the *trpE* coding sequence. Point mutations in the *trpE* reporter fusion (C20G/C22G) resulted in the mutant reporter P_BAD_-*trpE20*'-'*lacZ,* which was not substantially upregulated when wild-type RydC was produced (Fig. 7B). Because of the unusual pseudoknot structure of RydC (3, 31), mutations in the 3′ end of RydC have a dramatic impact on RydC stability (32); thus, we were not able to test a RydC compensatory mutant that would restore pairing to the *trpE20* mutant fusion. However, we did construct a second *trpE* fusion, P_BAD_-*trunc-trpE*'-'*lacZ,* which was truncated to remove the putative RydC binding site (Fig. 7B). This fusion was no longer activated by RydC at all. These observations suggest that sequences early in the *trpE* coding sequence are important for RydC-mediated increase in *trpE* translation.

## DISCUSSION

Over the years, many sRNAs have been discovered and characterized using both computational and experimental methods. Although target discovery of sRNAs still remains the rate-limiting step in sRNA characterization, many new techniques have been developed to overcome that obstacle. Some techniques take a purely computational approach to target prediction, including the target prediction algorithms we have included in SPOT (9–13) and others we have not included (33–43). Experimental techniques to identify bacterial sRNA targets have also expanded. Many of these techniques use affinity purification or coimmunoprecipitation approaches, with or without cross-linking (15, 17, 20, 44–46). To help streamline the process of sRNA target identification, the SPOT pipeline was constructed to be used in conjunction with other identification methods. In this study, we showed that the SPOT pipeline achieved ≥75% sensitivity and ≤50% false-positive rate when at least two methods converged on a prediction for the well-characterized sRNAs SgrS and RyhB (Fig. 2D and E). Expanding our analysis to other enterobacterial sRNAs, we found that the pipeline sensitivity was equal to or exceeded that of any individual method (average of 84% ± 8.5%; Fig. 3A and Fig. S2 in the supplemental material). As before, we found that correct identification by ≥2 methods occurred in the majority of instances (Fig. 3A). Furthermore, SPOT can be applied to the reverse situation where a user can search for potential sRNAs that regulate their UTR of interest. We found through these analyses that for 11 E. coli 5′ UTRs with ≥2 known interactions with sRNAs, the analysis gave an average sensitivity of 85% ± 24% (Fig. 3A).

To test the utility of SPOT in identifying novel sRNA-mRNA target interactions, we used it to predict targets of the poorly characterized sRNA RydC, which had been reported to regulate three genes: *yejA* (30), *cfa* (3), and *csgD* (31). Through SPOT analyses and filtering based on experimental data, we generated a list of putative RydC targets (Table 1). Reassuringly, SPOT identified the true RydC target, *cfa* mRNA, and correctly predicted the known binding site on this target (Table 1 and Data Set S1). The other two reported targets, *yejA* and *csgD,* were not identified by the SPOT computational pipeline, nor were these genes differentially regulated in our RydC pulse expression RNA-seq analyses (Data Set S2). Since no specific direct binding interactions were shown for RydC-*yejA* or RydC-*csgD*, we postulate that the previously observed regulation of these targets by RydC may be indirect. The SPOT pipeline also correctly identified two additional RydC targets, *pheA* and *trpE* (Table 1 and Fig. 5C, 6, and 7). RydC represses *pheA* translation, likely by a mechanism common to repressing sRNAs. Binding of RydC to sequences around the Shine-Dalgarno region would prevent ribosome binding and inhibit translation initiation. The mechanism of RydC-dependent activation of *trpE* appears to be more complex. The *trpE* gene is part of the *trpLEDCBA* operon responsible for L-tryptophan biosynthesis, which is regulated by both the *trpR* repressor and an attenuation mechanism (47). Depending on the availability of L-tryptophan, the ribosome either stalls at or moves quickly through Trp codons in the *trpL* ORF. When Trp is abundant, the ribosome rapidly completes translation of *trpL,* which prevents cotranscriptional formation of an antiterminator hairpin and allows formation of a transcription terminator just upstream of the *trpE* coding sequence. When Trp is limiting, ribosome stalling at the Trp codons allows formation of an antiterminator structure, which promotes transcription elongation into downstream Trp biosynthesis structural genes. While sequences within the *trpE* coding sequence have not been implicated in the Trp-dependent attenuation mechanism, it is possible that the sequences including the RydC binding site are responsible for yet another layer of regulation of these genes, perhaps at the level of translation. Alternatively, sequences in the *trpE* coding sequence could have long-range interactions with the upstream terminator or antiterminator sequences, and RydC binding could modulate those interactions.

Our study and evaluation of a combinatorial approach to identify mRNA targets of sRNAs of interest represent a step toward accelerating a rate-limiting step in sRNA characterization. The SPOT pipeline is able to streamline the process of running individual algorithms, which can take hours to days, by reducing the run times significantly for all four algorithms at once (under 2 h). Since the pipeline runs all four algorithms simultaneously, a more narrowed down, comprehensive list is generated, negating the need for manually selecting targets from individual algorithm runs. However, every method has drawbacks, and though SPOT is a powerful tool, it has limitations as well. For instance, a 50% false-positive rate (the average for well-characterized sRNAs analyzed in this study) is still high even though it is markedly better than the false-positive rates of predictions made by any single algorithm. As experimental approaches for sRNA-mRNA target identification continue to improve, the power and accuracy of SPOT’s combinatorial approach to sRNA target binding site predictions will likewise improve.

We were able to test the sensitivity and accuracy of SPOT for identification of sRNA-mRNA interactions (for example E. coli*/Salmonella* sRNAs) because of the availability of many defined and validated interactions. We do not have access to similar data sets from other bacteria, which limits our understanding of how SPOT will perform in predicting sRNA-mRNA interactions from other organisms, such as those with very different genomic GC content compared to E. coli and *Salmonella*. We note that each of the algorithms incorporated in SPOT uses a slightly different method to predict sRNA-mRNA interactions, including different ways to account for intrinsic structures of putative binding partners. Variations in RNA GC content would affect the predicted folding of each partner, and we would expect this to impact the output of prediction algorithms based on how they account for intrinsic RNA structure. More work is needed to optimize performance of computational algorithms for prediction of sRNA targets in diverse organisms, but SPOT provides a platform that can be customized to include or remove different computational algorithms, provided the code is freely available.

Another factor impacting the accurate prediction of sRNA binding sites by SPOT is the user-defined search window. The majority of early examples of sRNA-mediated regulation involved sRNAs binding in translation initiation regions of target mRNAs. Thus, most existing sRNA target prediction algorithms have default windows set to search around start codons. As more sRNA-mRNA interactions are validated and mechanisms of regulation studied, we and others have found increasing numbers of examples of sRNA-mRNA interactions that occur outside this window. Some of these interactions are primary or only interactions responsible for sRNA-mediated regulation of the mRNA, e.g., RydC-*cfa* mRNA (3), SgrS-*yigL* mRNA (27), which both involve mRNA sequences far upstream of the start codons. Yet other interactions involving mRNA sequences far from translation initiation regions represent secondary or auxiliary binding interactions that nevertheless play important roles in regulation (18, 26). For the sRNA SgrS, there are two binding sites for its interaction with *asd* mRNA (18), but SPOT was able to predict only the primary binding site. We expect that there are other examples where the algorithms have failed to identify alternate or additional binding sites. This is currently an area of development and once implemented, will serve as a valuable asset in identifying putative targets for an sRNA of interest.

Taken together, the combinatorial approach revealed two new targets, *pheA* and *trpE*, in the RydC regulon. Interestingly, both PheA and TrpE are involved in the chorismate metabolic pathway, with PheA using chorismate as a substrate in L-tyrosine/L-phenylalanine biosynthesis and TrpE for L-tryptophan biosynthesis. Interestingly, RydC repressed *pheA*, whereas it activated *trpE,* an unusual case, since both are involved in amino acid biosynthesis in divergent pathways. In the case for *trpE,* the mechanism of positive regulation is unique in that the base pairing interaction takes place 12 to 22 nt downstream of the start codon. RydC could possibly serve as an sRNA modulator of the biosynthetic pools of amino acids by activating/repressing *trpE* or *pheA* mRNA expression when necessary. As an aside, chorismate is also a substrate for production of the E. coli siderophore enterobactin, which is synthesized under iron limiting conditions. Mutations in *fur*, *tyrA*, *pheA*, or *pheU* resulted in increased enterobactin production, since the chorismate pools were used for enterobactin synthesis (48). These observations suggest that there may be conditions where RydC impacts the iron starvation stress response, perhaps forming a regulatory network that intersects with that of the well-characterized iron starvation stress response sRNA, RyhB. To better understand these potential connections, future work will be aimed at characterizing the regulators and conditions controlling synthesis of RydC.

With the implementation of the SPOT pipeline, combined with RNA-Seq and MAPS data, we were able to add to the RydC regulon and expand its network. Whether this regulatory network is exhaustive remains to be determined. We note that there were other RydC-mRNA binding interactions predicted by SPOT that were not analyzed further here. Moreover, there are additional sRNA-mRNA interactions predicted by SPOT for the other sRNAs that were run through the pipeline (Data Set S1), and it is likely that more bona fide interactions are among those predictions. All in all, we developed a streamlined method for sRNA-mRNA binding site predictions that leverages the strengths of many preexisting algorithms. We showed the robustness of SPOT for identification of true sRNA-mRNA interactions using well-characterized and poorly characterized sRNAs. We anticipate that SPOT will become a valuable tool for many investigators who have found interesting sRNAs and wish to identify potential mRNA targets for further characterization.

## MATERIALS AND METHODS

### Software pipeline.

A software pipeline was constructed in PERL to provide a single interface for running four sRNA-mRNA target prediction algorithms in parallel and collating their results (Fig. 1). Source codes for TargetRNA2 v2.01 (9), sTarPicker (10), IntaRNA v1.0.4 (12), and CopraRNA v 1.2.9 (13) were downloaded and installed on a multicore local server. The pipeline is comprised of four steps described briefly here.

1. Reference genome files are retrieved from RefSeq or local customized genome files can be used, provided they are in an appropriate RefSeq format (GBK file or PTT and FNA files).
2. Simultaneous searches are initiated for TargetRNA2, sTarPicker, and IntaRNA according to user-defined search parameters (e.g., window size, seed size, significance cutoffs). Optionally, if RefSeq IDs and corresponding sRNA sequences from related genomes are provided, a CopraRNA search is initiated.
3. The pipeline tracks the progress of each job, and once each search is completed, the raw results files are read into memory.
4. User-defined results filtering parameters are applied. The user can provide SPOT with a list containing, e.g., known mRNA binding coordinates, genes that are differentially expressed in sRNA-expressing cells compared to control cells, or operon data. SPOT will return only results relevant to the user-supplied list (described in more detail in Text S1 in the supplemental material). The raw results in memory are then collated into a unified report.

The collated results report includes Excel-formatted data tables, functional enrichment predictions for consensus mRNA targets, and binding plots. Examples of all output formats are shown in the SPOT Instructions (Text S1), and outputs for every sRNA examined in this study are shown in separate tabs in Data Set S1. SPOT produces a ranked list of predictions from algorithm runs as follows (with rank listed in the last column of each output table: (i) known (previously demonstrated) binding sites correctly predicted by ≥2 algorithms, (ii) known (previously demonstrated) binding sites correctly predicted by 1 algorithm, (iii) predictions that are inconsistent with known binding sites, (iv) predictions that are the same between ≥2 algorithms, (v) prediction only by CopraRNA, (vi) prediction only by TargetRNA or sTarPicker, and (vii) prediction only by IntaRNA. Both the collated results and individual search results can be downloaded once the job is complete. In addition, users can elect to have an e-mail notification sent when the job is complete. The pipeline also includes an option to rerun the result collation steps using different results filters. This enables users to make minor adjustments to the reporting of results without waiting for the individual searches to be rerun.

10.1128/mSphere.00561-18.1 TEXT S1 SPOT user manual. Download Text S1, PDF file, 1.3 MB. Copyright © 2019 King et al. 2019 King et al. This content is distributed under the terms of the Creative Commons Attribution 4.0 International license.

The SPOT program and installation instructions are available on GitHub (https://github.com/phdegnan/SPOT). In addition, an Amazon Web Service (AWS) cloud Amazon Machine Image (AMI) with all of the required software installed is available (search for SPOTv1). The SPOT user manual (Text S1) is also included in supplemental material.

### Generation of test data sets.

Known sRNA-mRNA interactions were collected from ecocyc.org (49), the literature, and experiments herein for 12 sRNAs with ≥4 confirmed targets: RyhB (b4451, RF00057), Spot42 (spf, b3864, RF00021), SgrS (b4577, RF00534), RybB (b4417, RF00110), FnrS (b4699, RF01796), GcvB (b4443, RF00022), OmrA (b4444, RF00079), CyaR (b4438, RF00112), MicA (b4442, RF00078), MicF (b4439, RF00033), DicF (b1574, RF00039), and RydC (b4597, RF00505) (Table S1). The confirmed sRNA-mRNA binding interactions were used as true-positive results to investigate the reliability and sensitivity of the pipeline.

10.1128/mSphere.00561-18.5 TABLE S1 Overview of confirmed and putative sRNA targets. Download Table S1, PDF file, 0.04 MB. Copyright © 2019 King et al. 2019 King et al. This content is distributed under the terms of the Creative Commons Attribution 4.0 International license.

In order to test CopraRNA, homologs for the 12 E. coli MG1655 sRNAs were identified in related genomes using Infernal (50). For all sRNAs excluding DicF, the genomes of Escherichia fergusonii ATCC 35469 (RefSeq accession no. NC_011740), Citrobacter koseri ATCC BAA-895 (NC_009792), and Salmonella enterica serovar Typhimurium LT2 (NC_003197) were queried with the Infernal algorithm and each covariance model. For the sRNA DicF, a phylogenetically restricted sRNA, E. coli O157:H7 strain Sakai (NC_002695) and E. coli strain APEC O1 (NC_008563) were queried. In cases where genomes encoded ≥1 prediction (e.g., OmrA), the prediction with the lowest E value was used.

In addition, we compiled a list of 85 E. coli sRNAs to investigate the ability of the pipeline to be used to predict mRNA-sRNA interactions using a putative mRNA target as the search query (Table S2). This includes 65 RefSeq annotated sRNAs (NC_000913.3), an additional 19 sRNAs annotated in ecocyc.org (49), and the sRNA IepX (51). Note that 552 additional predicted E. coli sRNAs, *cis* regulatory elements, and other putative RNAs corresponding to known RFAMs (*n* = 172) or identified from expression studies (*n* = 360) were not included (52, 53).

10.1128/mSphere.00561-18.6 TABLE S2 E. coli MG1655 known small RNAs. Download Table S2, PDF file, 0.05 MB. Copyright © 2019 King et al. 2019 King et al. This content is distributed under the terms of the Creative Commons Attribution 4.0 International license.

Finally, sRNA-mRNA interaction coordinates and the 5′ UTRs of 11 mRNAs with ≥2 known interacting sRNAs were collected from ecocyc.org (49): *csgD* (b1040, *n* = 5), *flhD* (b1892, *n* = 4), *ompA* (b0957, *n* = 3), *ompC* (b2215, *n* = 3), *ompF* (b0929, *n* = 2), *ompX* (b0814, *n* = 2), *phoP* (b1130, *n* = 2), *rpoS* (b2741, *n* = 4), *sdhC* (b0721, *n* = 3), *sodB* (b1656, *n* = 2), and *tsx* (b0411, *n* = 2).

### Media and reagents.

E. coli strains were cultured in lysogeny broth (LB) medium or on LB agar plates at 37°C, unless stated otherwise. For construction of reporter fusions by λ Red, recovery of recombinants was carried out on M63 minimal medium containing 5% sucrose, 0.001% L-arabinose (Ara), 0.2% glycerol, and 40 μg/ml 5-bromo-4-chloro-3-indolyl-β-D-galactopyranoside (X-Gal). For β-galactosidase assays, bacterial cells were grown in tryptone broth (TB) medium supplemented with 100 μg/ml ampicillin (Amp) overnight at 37°C and then subcultured in TB broth containing 100 μg/ml ampicillin (Amp) with 0.002% L-arabinose. Where necessary, media were supplemented with the following antibiotics and concentrations: 100 μg/ml ampicillin (Amp), 25 μg/ml chloramphenicol (Cm), and 25 μg/ml kanamycin (Kan). Expression of RydC was induced with either 0.1 or 0.5 mM isopropyl-β-D-1-thiogalactopyranoside (IPTG) from the P_LlacO-1_ promoter.

### Strain construction.

Strains and plasmids used in this study are listed in Table S3. All strains used in this study are derivatives of E. coli K-12 strain MG1655. Oligonucleotide primers and 5′-biotinylated probes used in this study are listed in Table S4 and were all acquired from Integrated DNA Technologies (IDT). Chromosomal mutations were made by λ Red recombination (54, 55), and marked alleles were moved between strains by P1 *vir* transduction (56). PCR products were generated using the Expand high-fidelity PCR system (Sigma-Aldrich, St. Louis, MO) according to the manufacturer’s instructions. All mutations were verified by amplifying PCR fragments using GoTaq polymerase (Promega, Madison, WI) and sequencing.

10.1128/mSphere.00561-18.7 TABLE S3 Strains and plasmids used in this study. Download Table S3, PDF file, 0.1 MB. Copyright © 2019 King et al. 2019 King et al. This content is distributed under the terms of the Creative Commons Attribution 4.0 International license.

10.1128/mSphere.00561-18.8 TABLE S4 Oligonucleotides used in this study. Download Table S4, PDF file, 0.1 MB. Copyright © 2019 King et al. 2019 King et al. This content is distributed under the terms of the Creative Commons Attribution 4.0 International license.

The translational *lacZ* reporter fusions under the control of the P_BAD_ promoter were constructed by PCR amplifying a fragment of interest using forward and reverse primers containing 5′ homologies to P_BAD_ and *lacZ* (Table S3). PCR products were recombined into strain PM1205 using λ Red homologous recombination and counterselection against *sacB* as described previously (57). The fusions used in this study were inserted into the *lac* locus of PM1205. Some *lacZ* reporter fusions used in this study were constructed using the one-step recombination method (58).

Plasmids harboring mutated *rydC* alleles under the control of the P_LlacO-1_ promoter were constructed using the QuikChange II XL site-directed mutagenesis kit (Agilent Technologies, Santa Clara, CA) with oligonucleotides AKP59 (P_LlacO-1_-*rydC3*), AKP68 (P_LlacO-1_-*rydC5*), and AKP69 (P_LlacO-1_-*rydC345*) that contained mismatched bases at the desired locations and transformed into XL10-Gold Ultracompetent cells (Table S3).

### RNA-seq analysis.

E. coli K-12 MG1655 strain AK250 (Δ*rydC lacI^q+^*) harboring vector (pBR322) or P*_lac_*-*rydC* plasmid was grown to an OD_600_ of ∼0.5 in LB broth medium at 37°C and then induced with 0.1 mM IPTG for 10 min. The hot phenol method (59) was used to extract total RNA after 2 and 10 min of induction. Samples were then treated with Turbo DNase (Ambion) kit according to the manufacturer’s protocol and resolved by gel electrophoresis on 1.2% agarose gel to confirm the integrity of the 16S and 23S bands. rRNA removal, library construction, and sequencing were performed at the W. M. Keck Center for Comparative and Functional Genomics at the University of Illinois at Urbana-Champaign. rRNA was removed from 1 μg of total RNA using Ribozero rRNA Removal Meta-Bacteria kit (Illumina, Inc.), and the mRNA-enriched fraction was converted to indexed RNA-seq libraries (single reads) with the TruSeq Stranded RNA sample preparation kit (Illumina, Inc.). The prepared libraries were then pooled in equimolar concentrations and were quantified by qPCR with the Illumina-compatible KAPA Library Quantification kit (Kapa Biosystems) and sequenced for 101 cycles plus seven cycles for the index read on a HiSeq2000 using TruSeq SBS version 3 reagents. The output fastq files were generated using Casava 1.8.2 (Illumina) and analyzed with Rockhopper (21). Genes were considered differentially expressed in RydC pulse-expression strains if they met a significance cutoff (q-value) of ≥0.005 and a fold change value of >1.5 or <0.5. Some genes outside this range were studied because they met other criteria (e.g., prediction of a RydC-mRNA interaction by multiple algorithms).

### β-Galactosidase assays.

Bacterial strains were cultured overnight at 37°C (shaking) in TB medium containing 100 μg/ml Amp. After the cultures were allowed to grow overnight, the cultures were diluted 1:100 into fresh TB medium containing 100 μg/ml Amp and 0.002% Ara and cultured at 37°C. After the cultures reached an OD_600_ of 0.3, 0.1 or 0.5 mM IPTG was added to induce expression of the plasmids and grown for an additional hour until an OD_600_ of 0.5 to 0.6 was reached. All β-galactosidase assays were performed as described in previous protocols (60). In short, the samples were suspended in Z-buffer, with reactions conducted at 28°C with 4 mg/ml 2-nitrophenyl-β**-**D-galactopyranoside (ONPG) as a substrate and 1 M Na**_2_**CO_3_ to end the reaction.

### Data availability.

RNA-seq data were submitted to NCBI Gene Expression Omnibus (GEO) and are available under the accession number GSE121595.
