# Supplementary figures and images for: sRNA Target Prediction Organizing Tool (SPOT) Integrates Computational and Experimental Data To Facilitate Functional Characterization of Bacterial Small RNAs
Source: mSphere. 2019 Jan 30;4(1):e00561-18. doi: 10.1128/mSphere.00561-18 (PMC6354806; doi:10.1128/mSphere.00561-18)

Figure S1

A. RyhB

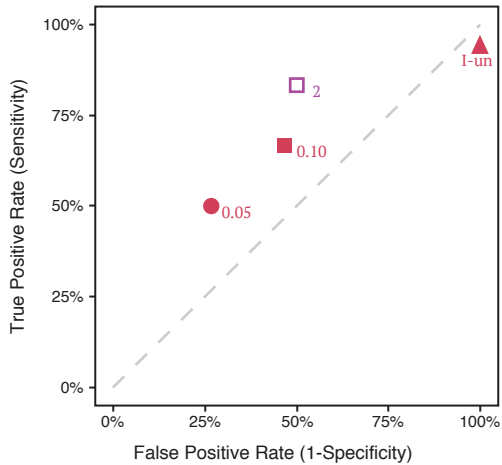

B. SgrS

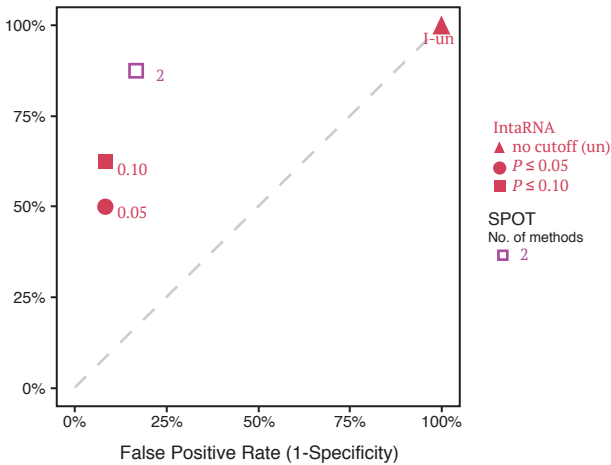

Supplement: FIG S1 [file mSphere.00561-18-sf001.pdf]

Figure S3

A.

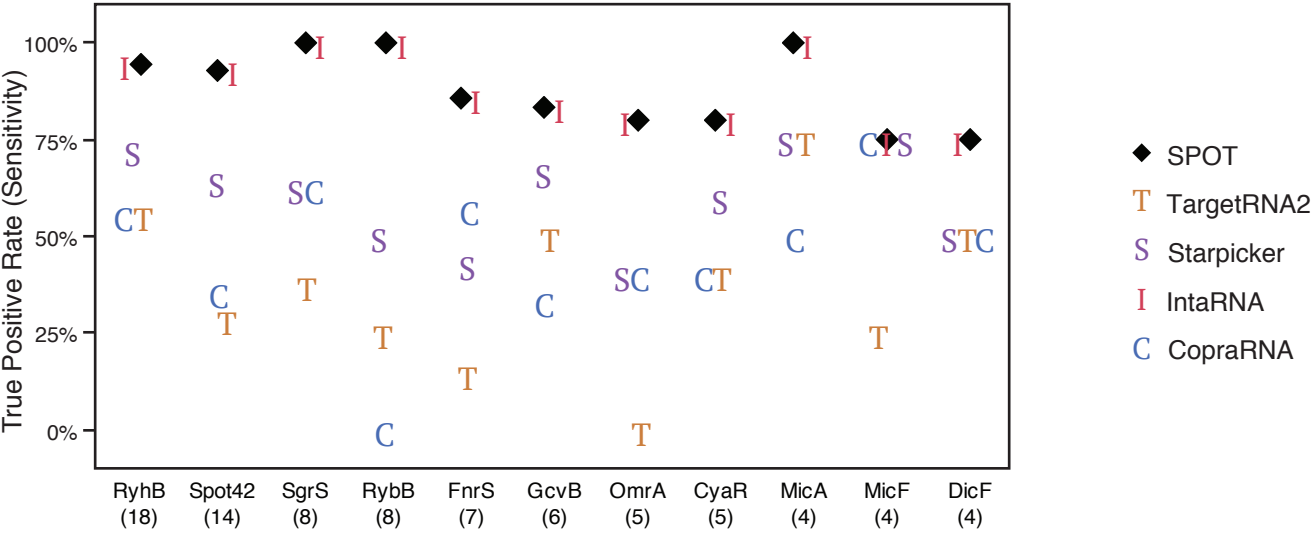

B.

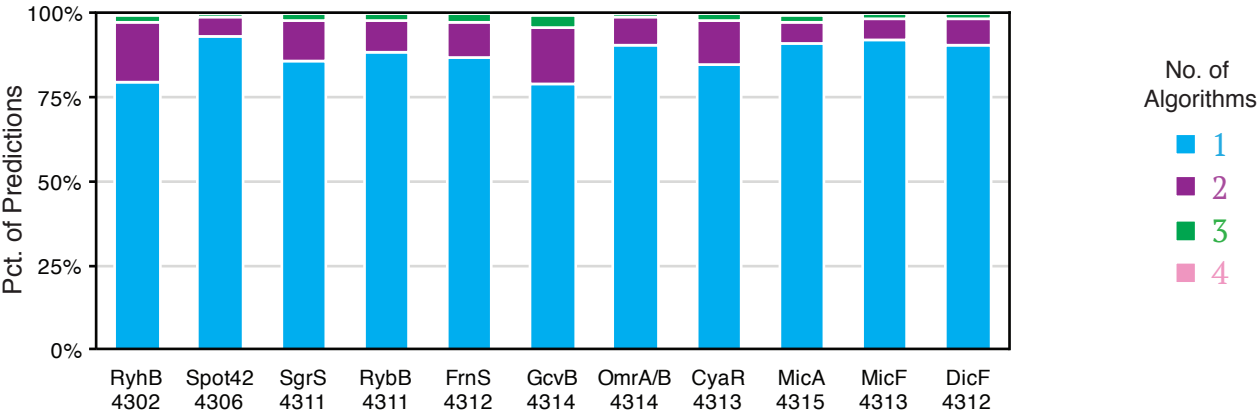

Supplement: FIG S3 [file mSphere.00561-18-sf003.pdf]
