# Supplementary material for: sRNA Target Prediction Organizing Tool (SPOT) Integrates Computational and Experimental Data To Facilitate Functional Characterization of Bacterial Small RNAs
Source: mSphere. 2019 Jan 30;4(1):e00561-18. doi: 10.1128/mSphere.00561-18 (PMC6354806; doi:10.1128/mSphere.00561-18)
Supplement: FIG S2 [file mSphere.00561-18-sf002.pdf]

Figure S2

A.

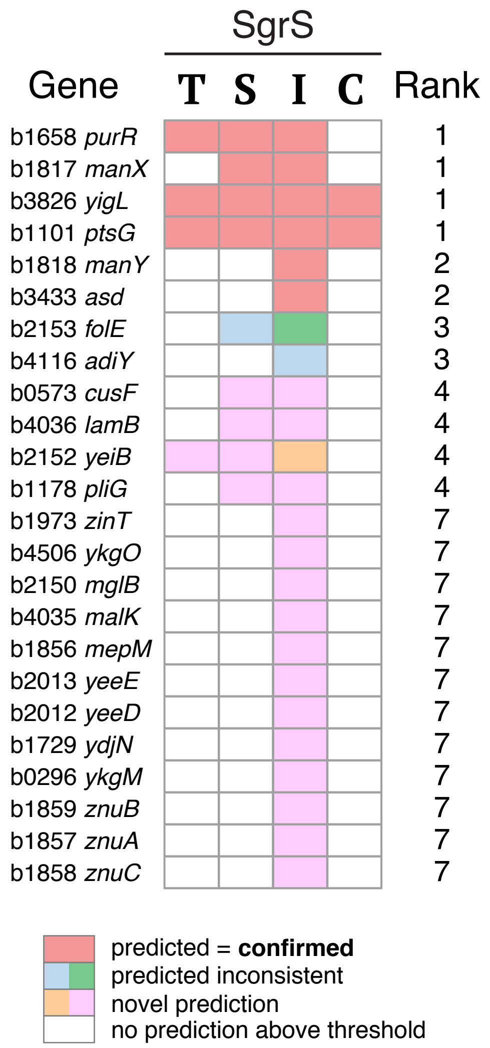

B.

cusF	Energy	Sig./ *P* value	sRNA start	sRNA stop	mRNA start	mRNA stop
Starpicker	-14.4	0.74	167	182	-151	-137
IntaRNA	-15.0	0.013	168	181	-150	-138

Starpicker

```
sRNA(Ec_sgrS)        167 uGUGUGACugaguauu  182
                          ||.|||| ||||||
Target(cusF_b0573)  -137 uCAUACUG-cucauau -151
```

IntaRNA

```
_____________-150__________-138
______________|______________|
___________5'-______________UUCAA...CAC-3'
______________AUACUC_GUCAUAC
______________||||||_||||:||
______________UAUGAG_CAGUGUG
3'-UUU...GGUGU______U_______UACUA...UAG-5'
_____________|______________|
_____________182____________167
```
