# Supplementary material for: sRNA Target Prediction Organizing Tool (SPOT) Integrates Computational and Experimental Data To Facilitate Functional Characterization of Bacterial Small RNAs
Source: mSphere. 2019 Jan 30;4(1):e00561-18. doi: 10.1128/mSphere.00561-18 (PMC6354806; doi:10.1128/mSphere.00561-18)
Supplement: TEXT S1 [file mSphere.00561-18-s0001.pdf]

SPOT
sRNA-target Prediction Organizing Tool

1. Rationale ........................................ 1
2. Installing SPOT ........................................ 3
3. Running SPOT - Quick Start ........................................ 5
4. Running SPOT ........................................ 6
5. Data input formats ........................................ 8
6. Data output formats ........................................ 11
7. Setup an AWS account ........................................ 14
8. Setup personal AWS interface on your laptop ........................................ 14
9. Starting an AWS instance ........................................ 15
10. Logging into you AWS instance ........................................ 21
11. References ........................................ 22

**Cite:** A.M. King, C.K. Vanderpool, and P.H. Degnan. sRNA-target Prediction Organizing Tool (SPOT) integrates computational and experimental data to facilitate functional characterization of bacterial small RNAs

## 1. Rationale

Computational approaches for sRNA target prediction have limitations but are relied upon to generate testable hypotheses for sRNA function. Some algorithms are available online or downloadable (e.g., TargetRNA2, IntaRNA), however these tools frequently yield distinct results, have different data output formats and default search parameters. Therefore, manually compiling results from these disparate tools and integrating the predictions with existing experimental data is not trivial. We have generated an innovative approach to streamline use of multiple existing sRNA target prediction algorithms and integrate predictions with experimental data to generate a unified set of target predictions. To this end, we have developed **SPOT** a flexible software pipeline that searches for sRNA-mRNA binding sites in parallel using separate search tools, collates the predictions, and integrates experimental data using customizable results filters.

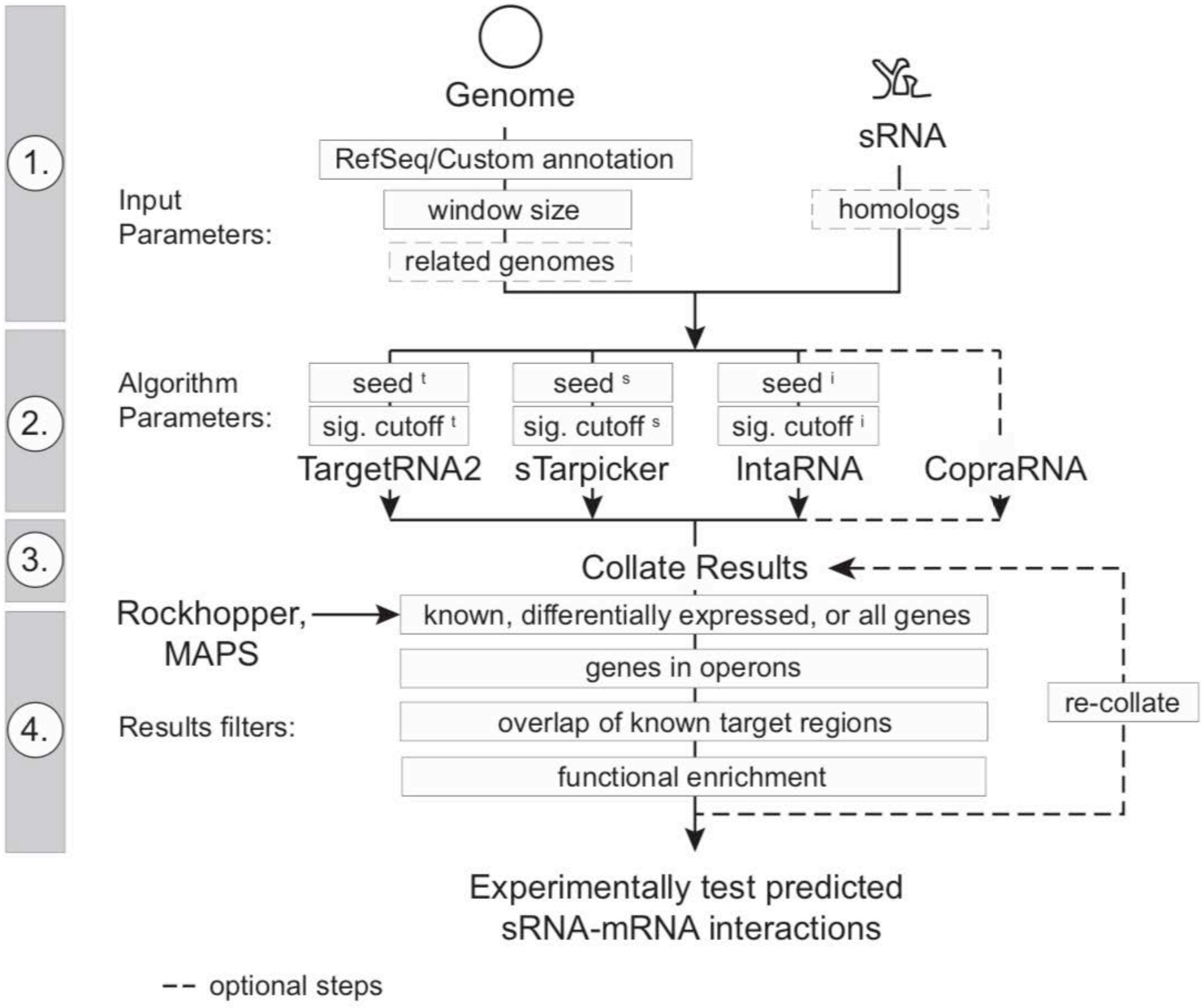


Figure 1. Schematic of **SPOT** pipeline analysis (King et al.)

## 2. Installing SPOT

**SPOT** is a PERL program that runs TargetRNA2, IntaRNA, StarPicker and CopraRNA in parallel, and collates the results to find consensus sRNA-mRNA targets (Figure 1). Furthermore, additional data types can be utilized to filter the results including expression differences, known binding sites, operon predictions and window size of possible binding sites.

As written the program can run on any Unix/Linux based system, however it has a number of dependencies. To facilitate its use we have set up an Amazon Web Service (AWS) cloud Amazon Machine Image (AMI) with all of the required software installed. Skip to sections 4-7 for setting up your own **SPOT** AMI. However, using the code available here you can set up and run **SPOT** on a local server.

First, download and install the following software tools and all of their dependencies according the authors' instructions:

- TargetRNA v2
- StarPicker
- IntaRNA v1.0.4
- CopraRNA v1.2.9

Several modifications were made to the StarPicker and IntaRNA code to accommodate demands of the pipeline.

Replace the following programs with those provided in the GitHub link. Modifications in the code are marked with ## comments and/or initials (PHD). Descriptions of edits made are listed briefly below.

StarPicker:

`sTarPicker_global2.pl` : changes made to input of command line arguments

IntaRNA v1.0.4:

`add_GI_genename_annotation.pl` : distinguish GeneIDs vs GI Nos
`get_refseq_from_ftp.pl` : Replacement code for get_refseq_from_ftp.sh
`IntaRNA_wrapper.pl` : Option added to use local GenBank files, use get_refseq_from_ftp.pl
`rerun_enrichment.pl` : code snippet re-running enrichment analysis from IntaRNA_Wrapper.pl
`termClusterReport.pl` : code modified to handle GeneIDs vs GI Nos

CopraRNA v1.2.9:

`get_refseq_from_ftp.pl` : Replacement code for get_refseq_from_ftp.sh
`termClusterReport.pl` : code modified to handle GeneIDs vs GI Nos
`get_CDS_from_gbk.pl` : code modified to skip and flag GenBank files not present in kegg2refseqnew.csv list

Note: D3 Javascript libraries may or may not be accessible using existing framework to generate functional enrichment heatmaps (http://d3js.org/d3.v3.min.js). If problems are encountered, it is possible to edit the master html files in IntaRNA and CopraRNA to use a local version of d3.v3.min.js .

Be sure all programs are added to the user path and all path references in StarPicker, IntaRNA, and CopraRNA match your system installation.
The statistics program R is installed as a requirement for IntaRNA and CopraRNA. As such add the following two packages:

- RColorBrewer
- gplots

```
$ sudo R
> install.packages(c('RColorBrewer', 'gplots'))
```

Some of the output from the **SPOT** program will be written in an `xlsx` format using the Excel Writer PERL module:

- Excel-Writer-XLSX-0.98

```
$ sudo cpan Excel::Writer::XLSX
```

Most existing Unix/Linux installations should have `sendmail` installed. If not, install the appropriate package

- sendmail

```
$ sudo apt install sendmail-bin
```

**SPOT** can work with local copies of genomes and annotations. However, to access genomes from NCBI install the `efetch` program from the Entrez Direct (`edirect`) toolkit.

- edirect

Retrieve and decompress the **SPOT** directory from GitHub containing core pipeline script and its additional required support PERL scripts.

- SPOT

Make sure **SPOT** and all of the programs are in your user path. Modify the core pipeline script with the absolute path locations for TargetRNA2, IntaRNA, StarPicker and CopraRNA, and other support PERL scripts.

## 3. Running SPOT - Quick Start

**SPOT** is a pipeline script that when run without arguments will print all of the possible program options:

```
$ spot.pl
Usage ./spot.pl
Input parameters:
-r    Fasta file of sRNA query
-a    RefSeq Accession number (assumes any local files have RefSeq
      number as their prefix)
...
```

The minimum data required for a **SPOT** search are:

1. A fasta file of the small RNA sequence
2. A RefSeq genome accession number

```
$ spot.pl -r sgrS.fasta -a NC_000913
```

This will initiate a job using the SgrS as the sRNA query and the *E. coli* str. K12 (NC_000913) as the reference genome. Progress of the search will be printed to the screen. Run time will depend on the number of processors available as each search tool is distributed to a separate sub process. By default CopraRNA **is not run** unless specifically requested.

## 4. Running SPOT

**SPOT** has an array of actions that control the input, algorithm parameters, and results filtering.

```
$ spot.pl
Usage ./spot.pl
Input parameters:
      -r    Fasta file of sRNA query
      -a    RefSeq Accession number (assumes any local files have RefSeq
            number as their prefix)
      -o    output file prefix (default = TEST)
      -g    Use local GenBank or PTT&FNA files for all Programs? (default = N
            use latest from GenBank, CopraRNA cannot use local files)
      -n    Other genome RefSeq ids for CopraRNA listed in quotes '' ,
            current max is 5 genomes (default ='')
      -m    Multisequence sRNA file for each genome in CopraRNA list
             (default ='')
      -x    Email address for job completion notification (default ='')

Algorithm parameters:
      -u    Number of nt upstream of start site to search (default = 60)
      -d    Number of nt downstream of start site to search (default = 60)
      -s    seed sizes for I, T, S e.g., '6 7 6' (defaults TargetRNA = 7,
            IntaRNA & Starpicker = 6)
      -c    P/Threshold value Cutoff for T, S, I e.g., '0.5 .001 un'
      (defaults Target = 0.05, Starpicker = 0.5, IntaRNA = top)

Results Filters:
      -b    Number of nt upstream of start site to filter results
             (default = -20)
      -e    Number of nt downstream of start site to filter results
             (default = 20)
      Note: -b and -e ignored if using a list (-l) or Rockhopper
            results (-t)
      Note: Set -b and -e to -u and -d to get all possible matches in
            results
      -l    List of up and/or down regulated genes, include binding coord if
            known e.g.,
      b1101\tdown\n
      b3826\tup\tsRNA_start\tsRNA_stop\tmRNA_start\tmRNA_stop\n
                                OR
      -t    transcriptome expression file from Rockhopper *_transcripts.txt
      -f    Rockhopper fold change cutoff (default = 1.5)
      -q    Rockhopper q value cutoff (default = 0.01)
      -k    Rockhopper Expression cutoff value (default = 100)
      -p    Operon file from DOOR-2 (http://csbl.bmb.uga.edu/DOOR/index.php)
             (optional)
      -w    Report all genes even if List or Rockhopper provided?
             (default = No)
      -y    Exclude target predictions by only 1 method? (default = Yes)
            Note: Does not apply to genes on List or significantly expressed
            from Rockhopper
      -z    Skip sRNA-mRNA detection steps, and just re-analyze data [Yy]es
            (default = No) (Run in the same directory & requires original
            results files from each program)
```

Given the time **SPOT** runs can take it is recommended to use a queueing tool on large distributed servers (`qsub, slurm`). Alternatively, on the AWS server, laptop or other smaller computers it is recommended to use `screen` to ensure that jobs are not prematurely aborted if the user account is logged out of.

```
$ screen -L spot.pl -r sgrS.fasta –a NC_000913
```

Four test datasets and precomputed output files are included in the folder `example_files`. The following examples correspond to the four provided test datasets.

**test01 -** Examine entire *E. coli* str. K12 genome for SgrS sRNA target mRNAs. This folder only has the sRNA sequence in a `fasta` file, uses the individual program default SEED size and significance settings and retrieves the genome sequence for *E. coli* from GenBank. The final option is to have an email sent to the user after the job has completed.

```
$ cd test01
$ ls
sgrS.fasta
$ spot.pl -r sgrS.fasta –o stringent –a NC_000913 –x username@email.edu

=========Prepping RefSeq Files====================
[Thu Aug 23 23:14:27 UTC 2018]
...
```

**test02 -** Examine *E. coli* str. K12 genome for SgrS sRNA target mRNA matches among a set of defined differentially expressed genes (`sgrS_diff.txt`). In this case the user has a `fasta` file and a traditional GenBank protein translation file (`PTT`). The user also indicates a larger window size 150 nt upstream of the CDS start position and 100 nt downstream to search for binding sites.

```
$ cd test02
$ ls
sgrS.fasta
sgrS_diff.txt
NC_000913.fna
NC_000913.ptt
$ spot.pl -r sgrS.fasta –l sgrS_diff.txt –u 150 –d 100 –c '0.5 0.001 un' –o
relaxed –a NC_000913 –g Y
```

*Note:* `PTT` files can be easily generated in Excel. Allowing for *customization* of gene annotations and subsequent analyses. A script included with **SPOT** is `fnaptt2gbk.pl` which can be used to generate GenBank files using the genome `PTT` and `fasta` files as inputs. However, always make sure that MAC or DOS line breaks are converted into UNIX line breaks.

**test03 - SPOT** was designed to allow re-analysis of existing results. This example code block is run in a folder containing the results of **test02**'s search. In this case even though the upstream/downstream region searched was 150nt and 100nt, the reanalysis eliminates any binding sites found outside of 50nt upstream and 30nt downstream. This search also does not use the list of differentially expressed genes.

```
$ cd test03
$ ls
sgrS.fasta
sgrS_diff.txt
NC_000913.fna
NC_000913.ptt
...
$ spot.pl -r sgrS.fasta -u 150 -d 100 -c '0.5 0.001 un' -o changed_50_30 -a
NC_000913 -g Y -b -50 -e 30 -z Y
```

**test04 – SPOT** can also be run using a `*transcript.txt` file generated by the RNAseq analysis program Rockhopper directly (instead of list as in example **test02**). In this example default expression cutoffs are used, however these can be specified by the user. In addition, when provided a set of sRNA homologs and target genomes CopraRNA can be run. In these instances only genomes in RefSeq can be used. Custom genome annotations cannot be utilized.

```
$ cd test04
$ ls
NC_000913_SgrS_transcripts.txt
sgrS.fasta
sgrS_homologs.fasta
$ spot.pl -r sgrS.fasta -t NC_000913_SgrS_transcripts.txt -o express -a
NC_000913 -m sgrS_homologs.fasta -n 'NC_002695 NC_011740' -u 150 -d 100
```

When the jobs have completed compare your results to the files in the corresponding `_results` folder.

## 5. Data input formats

*sRNA fasta file* – DNA sequence of sRNA in a standard fasta file. File extension does not matter (`.fasta, .fa, .fna, .frn, .ffn`)

*RefSeq ID* – Standard RefSeq IDs can be used and GenBank files (`.gbff`) will be retrieved using `efetch`. Program will retrieve additional replicons (e.g., plasmids) or scaffolds associated with the provided RefSeq IDs, however, the search will only be

carried out on the file with a name corresponding to the input RefSeq ID. By default the `.gbff` is renamed to a `.gb` file, and `.fna` and `.ptt` files are generated.

_Local Files_ – Different combinations of local files can be used. They all **must** have the same prefix and end in the following suffixes:

| | |
|---|---|
| `.fna` | Genome fasta sequence |
| `.ptt` | Protein translation table – gene annotation |
| `.gb` or `.gbk` | Genbank file |

Files ***without*** these suffixes will be ignored. All must have Unix linebreaks and the `.ptt` file must be tab separated. Allowed input combinations include:

| | **.fna** | **.ptt** | **.gb or .gbk** | **Status** | **Action** |
|---|---|---|---|---|---|
| 1. | √ | √ | √ | okay | Start run |
| 2. | √ | √ | | okay | Make `.gb` file, start run |
| 3. | √ | | √ | okay | Make `.ptt` file, start run |
| 4. | | √ | √ | okay | Make `.fna` file, start run |
| 5. | | | √ | okay | Make `.fna` and `.ptt` file, start run |
| 6. | √ | | | bad | Abort run |
| 7. | | √ | | bad | Abort run |

_`.ptt` Files_ – This is a legacy GenBank annotation format. However, the StarPicker algorithm used here requires this format. This format is very easy to generate in Excel and can allow users of **SPOT** to customize their annotations. See example:

| | | | | | | | | |
|---|---|---|---|---|---|---|---|---|
| Escherichia coli str. K-12 substr. MG1655, complete genome - 1..4641652 | | | | | | | | |
| 4141 proteins | | | | | | | | |
| Location | Strand | Length | PID | Gene | Synonym | Code | COG | Product |
| 190..255 | + | 21 | | thrL | b0001 | - | - | thr operon leader peptide |
| 337..2799 | + | 820 | | thrA | b0002 | - | - | Bifunctional aspartokinase |
| 2801..3733 | + | 310 | | thrB | b0003 | - | - | homoserine kinase |
| 3734..5020 | + | 428 | | thrC | b0004 | - | - | L-threonine synthase |

*Note:* As indicated above, customization of `PTT` files allows users to correct or change annotations based on new data. Furthermore, by modifying `PTT` files **RNAs can be included** in the annotation. First, this allows for sRNA – RNA interactions to be identified. Second, this approach was used in the manuscript to perform a 'reverse' search. For a 'reverse' search the `PTT` file is edited to ONLY include the known sRNAs. Then, the user supplies the UTR or putative sRNA binding region to **SPOT** as a `fasta` file if it were the sRNA. 'Reverse' searches cannot use CopraRNA and as sRNAs do not have GI numbers and may not have GeneIDs - no functional enrichment plots will be produced. This may result in several warnings when the **SPOT** is run, however it should not influence the final composite predictions.

*Differentially expressed genes* – Lists of differential genes can be formatted as tab separated files one of two ways. **DO NOT include a header line.**

Simple:

| Locus | Expression |
|---|---|
| b1101 | down |
| b3826 | up |

With known binding sites:

| Locus | Expression | sRNA_start | sRNA_stop | mRNA_start | mRNA_stop |
|---|---|---|---|---|---|
| b1101 | down | 168 | 187 | -30 | -9 |
| b3826 | up | 168 | 187 | -96 | -76 |

*Rockhopper `*transcript.txt` files* – **SPOT** can read default output files of Rockhopper from simple pairwise RNAseq experiments. Files generated with the verbose output option in Rockhopper cannot be read. Files should have 12 columns including the normalized expression values for the treatment and control, the *q* Values and the estimated fold-change.

*sRNA Multisequence Fasta Files* – If running CopraRNA, the sRNA file must conform to expectations of the CopraRNA program:

1. RNA sequences must have Us instead of Ts
2. The sequence names must correspond to the individual genome RefSeq IDs
3. Must include the focal genome sRNA sequence

*Operon file from DOOR-2* – sRNAs binding sites may lie within a single gene or its UTR, however, bacterial genomes are organized into operons. Therefore, sRNAs may affect adjacent genes. If a list or `*transcript.txt` file is provided to identify focal genes in combination with an operon file (`*opr`), genes in the same transcriptional unit will be included in the final output. For example, in a 4 gene operon A, B, C, D, if only B was significantly differentially expressed, sRNA-mRNA predictions for genes A, C, D will be included in the output files too. Files conforming to those provided by the DOOR[2] Database of Prokaryotic Operons must be used. **A header line IS expected.**

| OperonID | GI | Synonym | Start | End | Strand | Length | COG_ number | Product |
|---|---|---|---|---|---|---|---|---|
| 2996 | 16127995 | b0001 | 190 | 255 | + | 21 | - | thr operon leader peptide |
| 2996 | 16127996 | b0002 | 337 | 2799 | + | 820 | COG0527E | Fused aspartokinase I and homoserine dehydrogenase I |

*Note:* Like `*ptt` files, `*opr` files can be easily generated from other resources using Excel or with other text tools.

## 6. Data output formats

Data from each individual algorithm is preserved in the output folder for manual investigation.

`TargetRNA2_*txt` = TargetRNA2 Primary report
`*.output` = Starpicker Primary report
`intarna_websrv_table_truncated.csv` = IntaRNA Primary report
`*_ hIntaRNA.csv` = CopraRNA Primary report

**SPOT** generates several output files for further analysis:

*XLSX file* – Primary file containing consensus table of sRNA-mRNA predictions from the 3 or 4 tools used in the run. File name prefix corresponds to run output prefix that was assigned (`-o , default= TEST`).

- Sheet 1 (`*_complete.txt`) shows the aligned predictions, *p* values, and coordinates for the predicted interaction for each gene.

<table>
<tr><th></th><th>A</th><th>B</th><th>C</th><th>D</th><th>E</th><th>F</th><th>G</th><th>H</th><th>I</th><th>J</th><th>K</th><th>L</th><th>M</th><th>N</th><th>O</th><th></th></tr>
<tr><td>1</td><td>Locus</td><td>Gene</td><td>T-Energy</td><td>T-Pvalue</td><td>T-sRNA_start</td><td>T-sRNA_stop</td><td>T-mRNA_start</td><td>T-mRNA_stop</td><td>T-Structure</td><td>S-Energy</td><td>S-Pvalue</td><td>S-sRNA_start</td><td>S-sRNA_stop</td><td>S-mRNA_start</td><td>S-mRNA_stop</td><td>S-Structure</td></tr>
<tr><td>2</td><td>b1101</td><td>ptsG</td><td>-10.51</td><td>0.018</td><td>167</td><td>187</td><td>-29</td><td>-9</td><td>Ec_sgrS 187 3' U-GUGGUUAUGAGUCAGUGUGU 5' 167<br>| |||| ||||||||  :|||<br>ptsG -29 5' AGCACCCAUACUCAGGAGCAC- 3' -9<br>gene product:fused glucose-specific PTS enzymes: IIB component/IIC component; PTS system, glucose-specific IIBC component</td><td>-20.8</td><td>0.004</td><td>169</td><td>187</td><td>-28</td><td>-8</td><td>sRNA(Ec_sgrS) 169 --uguga<br>||.<br>Target(ptsG_b1101) -8 ucacgag</td></tr>
<tr><td>3</td><td>b0060</td><td>polB</td><td>-9.86</td><td>0.025</td><td>151</td><td>169</td><td>-2</td><td>17</td><td>Ec_sgrS 169 3' UGUACUACGUCCGUUCAGU 5' 151<br>:|:||: |||||||:||:<br>polB -2 5' GCGUGGCGCAGGCAGGUUU 3' 17<br>gene product:DNA polymerase II</td><td></td><td></td><td></td><td></td><td></td><td></td><td></td></tr>
</table>

- Sheet 2 (`*_summary.txt`) has the counts predicted by each gene, and a summary letter and ranking based location and on the number of algorithms that found the same prediction.

**A** Prediction overlaps a known binding site
**B→E** Predictions that are not coincident with a known binding site when one was provided for that gene. Shared letters overlap the same site.
**F→I** Predictions when no known binding site was provided. Shared letters overlap the same site.

| | A | B | C | D | E | F | G | H | I | J | K | L | M | N |
|---|---|---|---|---|---|---|---|---|---|---|---|---|---|---|
| 1 | Locus | Gene | Target RNA2 | Star | IntaRNA | Count _ALL | TargetRNA2_-20_20 | Star_-20_20 | IntaRNA_-20_20 | Count_-20_20 | TargetRNA 2_M | Star_M | IntaRNA_ M | Rank |
| 2 | b1101 | ptsG | 1 | 1 | 1 | 3 | 1 | 1 | 1 | 3 | F | F | F | 4 |
| 3 | b0060 | polB | 1 | | 1 | 2 | 1 | | 1 | 2 | F | | F | 4 |

*_summary.pdf file* – This file has a R generated plot that corresponds to Sheet 2 (`*_summary.txt`) which can be imported to Illustrator.

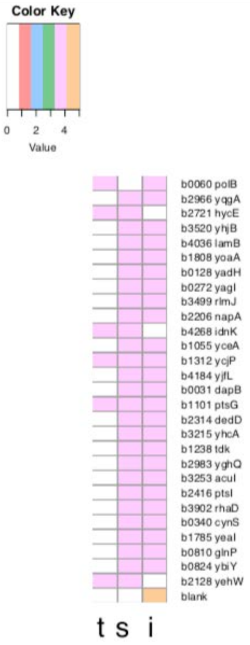


*COLLATED_RESULTS folder* – This folder contains plots generated based on IntaRNA tools showing the localization of binding sites of the mRNA and sRNA as `*pdf, *png` and `*ps` files.

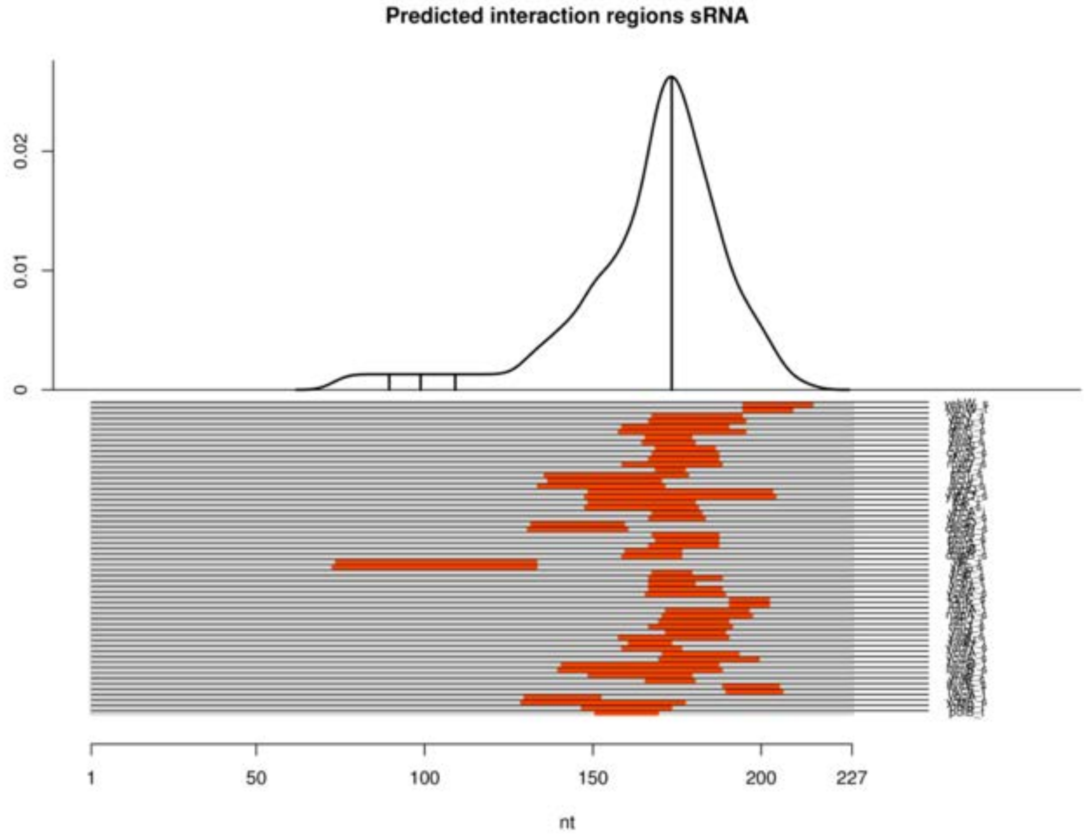

In addition, a functional enrichment heatmap is included as a `*pdf` file similar to those individually provided by IntaRNA and CopraRNA - however it represents the collated results.

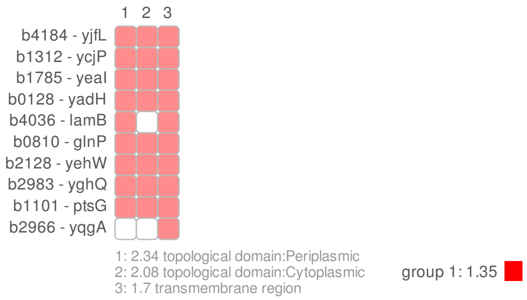


It is possible given the number and type of consensus predictions that are made, that no functional enrichment is produced. See the program file log, for possible comments errors that were noted while running the program.

Example result files for the sRNA SgrS and corresponding test datasets are available with the **SPOT** software distribution.

## 7. Setup an AWS account

Navigate to the new account setup page:
https://portal.aws.amazon.com/billing/signup#/start

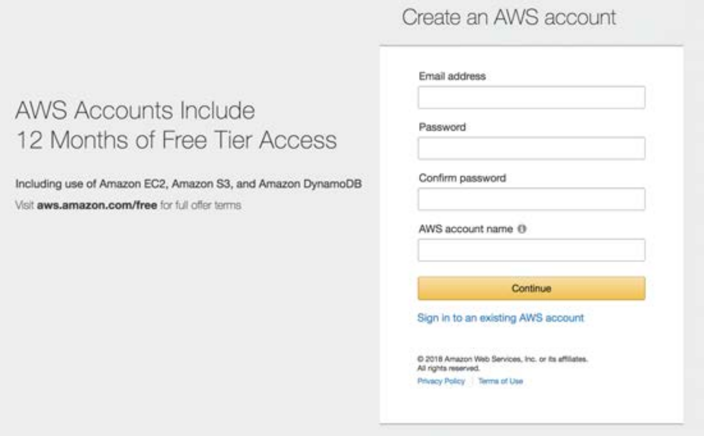


For now, set up your home region as "**U.S. East (W. Virginia)"** later you can switch this as necessary.

Unfortunately, when setting up an account you will need a credit card number

**Input Education credit -** Depending on your application it may be possible to apply for education credits to defray the cost of the AWS server time:

https://aws.amazon.com/education/awseducate/

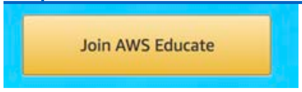


## 8. Setup personal AWS interface on your laptop

***People with MACs:***

Terminal will already be installed /Applications/Utilities

Download & Install **XQuartz** if not already installed
http://xquartz.macosforge.org/landing/

Download & Install **Cyberduck** https://cyberduck.io/?l=en

***People with PCs:***

Download & Install **PuTTY**
http://www.chiark.greenend.org.uk/~sgtatham/putty/download.html

Download & Install **xMing**
http://sourceforge.net/project/downloading.php?group_id=156984&filename=Xming-6-9-0-31-setup.exe

How to setup **xMing** : http://www.geo.mtu.edu/geoschem/docs/putty_install.html

Download & Install **WinSCP** http://winscp.net/eng/download.php
or
Download & Install **Cyberduck** https://cyberduck.io/?l=en

## 9. Starting an AWS instance

For in-depth instructions regarding starting an AWS instance please see:

https://docs.aws.amazon.com/AWSEC2/latest/UserGuide/launching-instance.html

1. After making and logging into your AWS account find your way to the EC2 (Elastic Computing Cloud) page. You can find it under "Services" menu on the upper left-hand corner of the page:

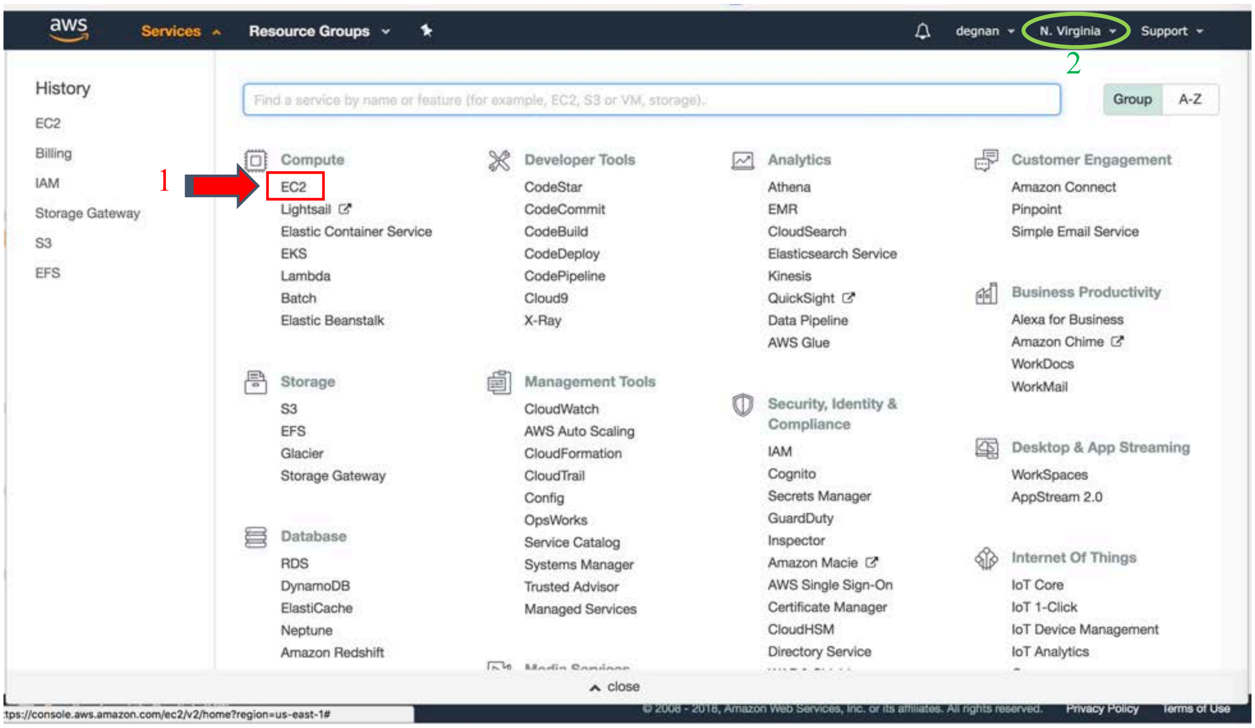


https://console.aws.amazon.com/ec2/v2/home?region=us-east-1#Home:

2. Make sure your home region as "**U.S. East (W. Virginia)**". Your region is indicated in the upper right-hand corner of the page (circled above)

3. On the right-hand side bar under "**IMAGES**" select "**AMIs**"

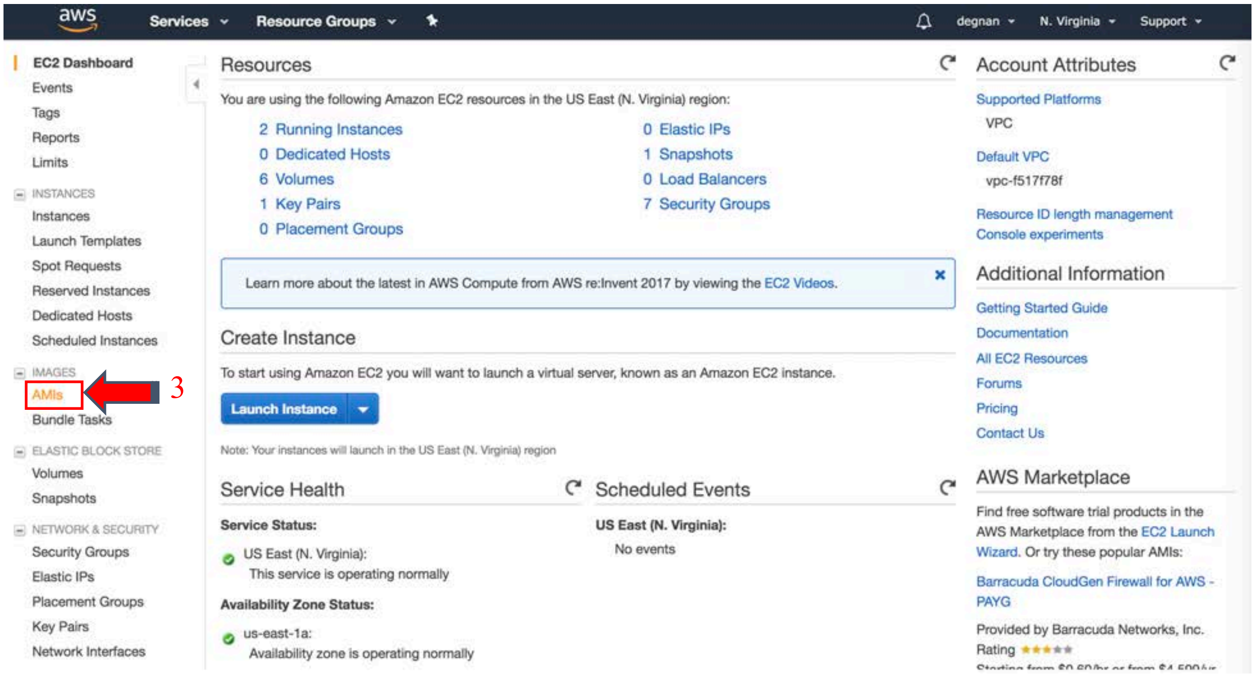


4. In the search bar switch from "Owned by me" to "**Public images**" and search for "**SPOTv1**"

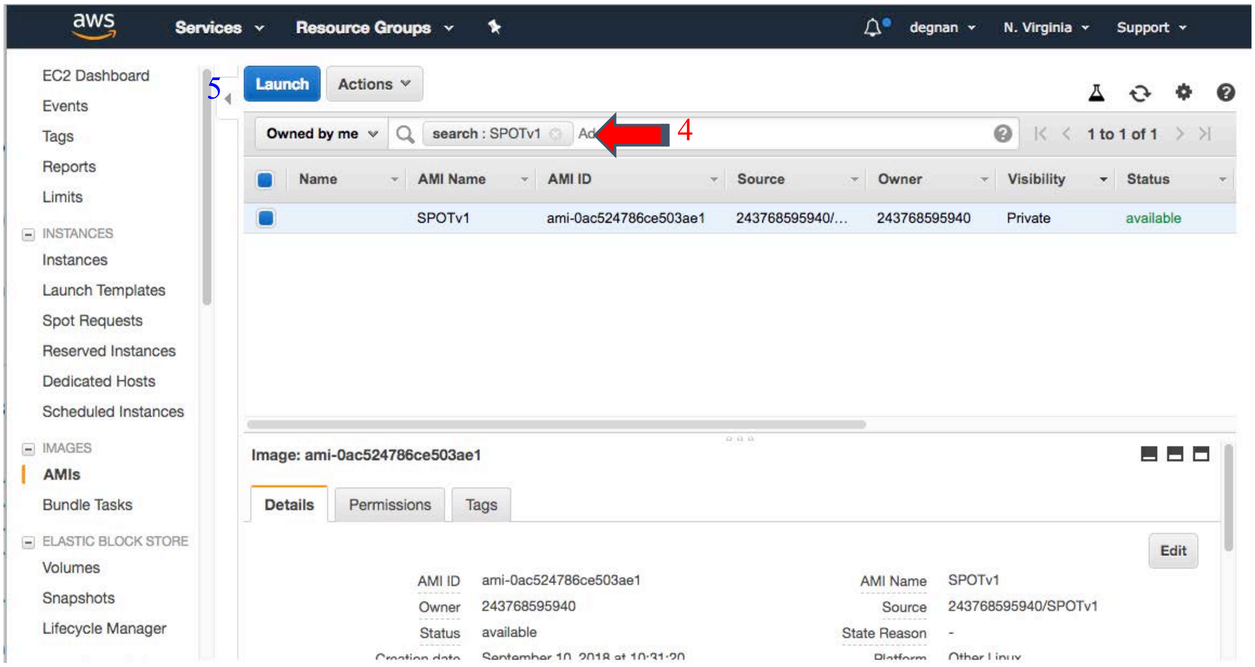


5. Select the blue "**Launch**" button

6. Now you are on AWS “Step 2: Choose and Instance Type” – Select your computer:

> **t2.micro** is the only free option, however it is maxed out at 1GiB of RAM, 1 processor and 30GiB of storage. *Very slow*
>
> **m5.2xlarge** 8 virtual processors, 64 GiB of RAM

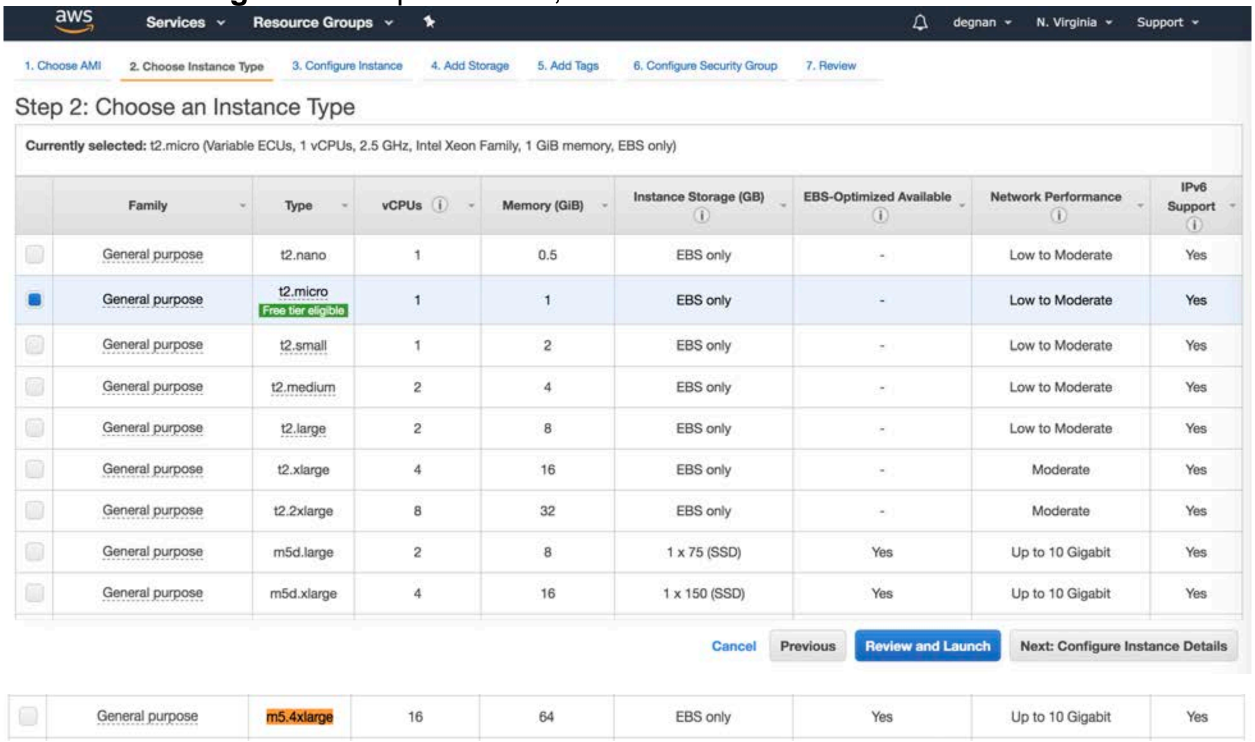


7. Select “Next: Configure Instance Details” button on bottom-right

8. On “Step 3: Configure Instance Details” page – ***leave defaults as-is***

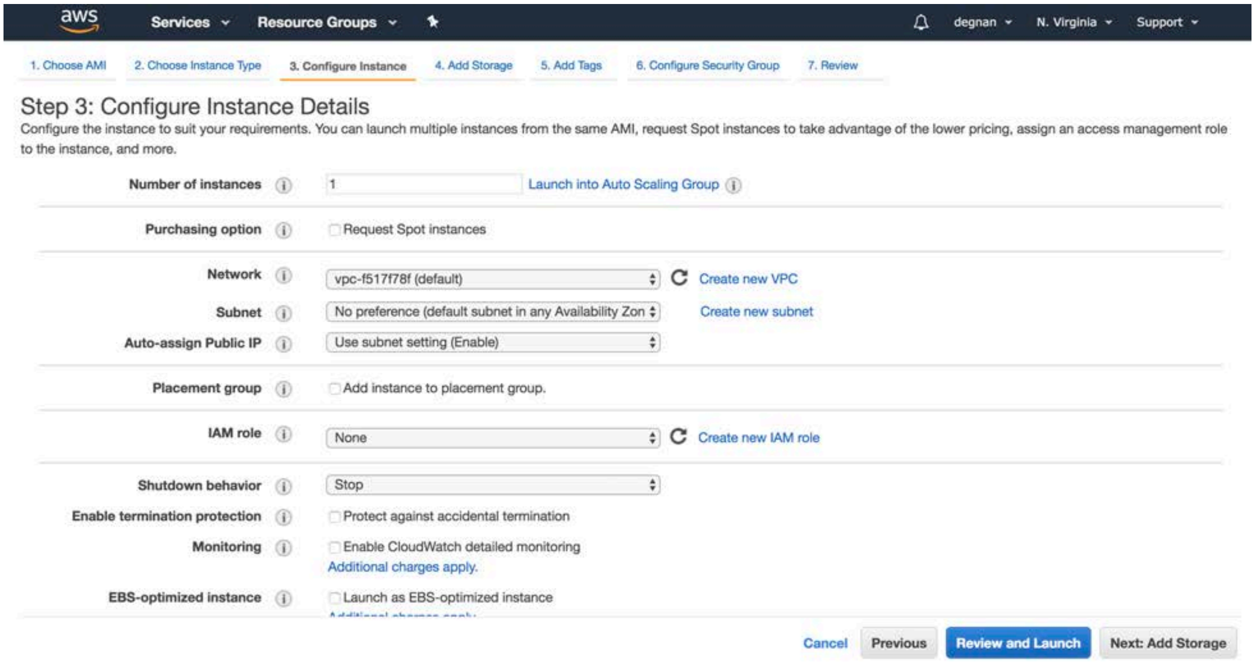

9. Select the “Next: Add Storage” button on bottom-right, to move to the next step

10. On the “Step 4: Add Storage” adjust local disk size to 30 GiB

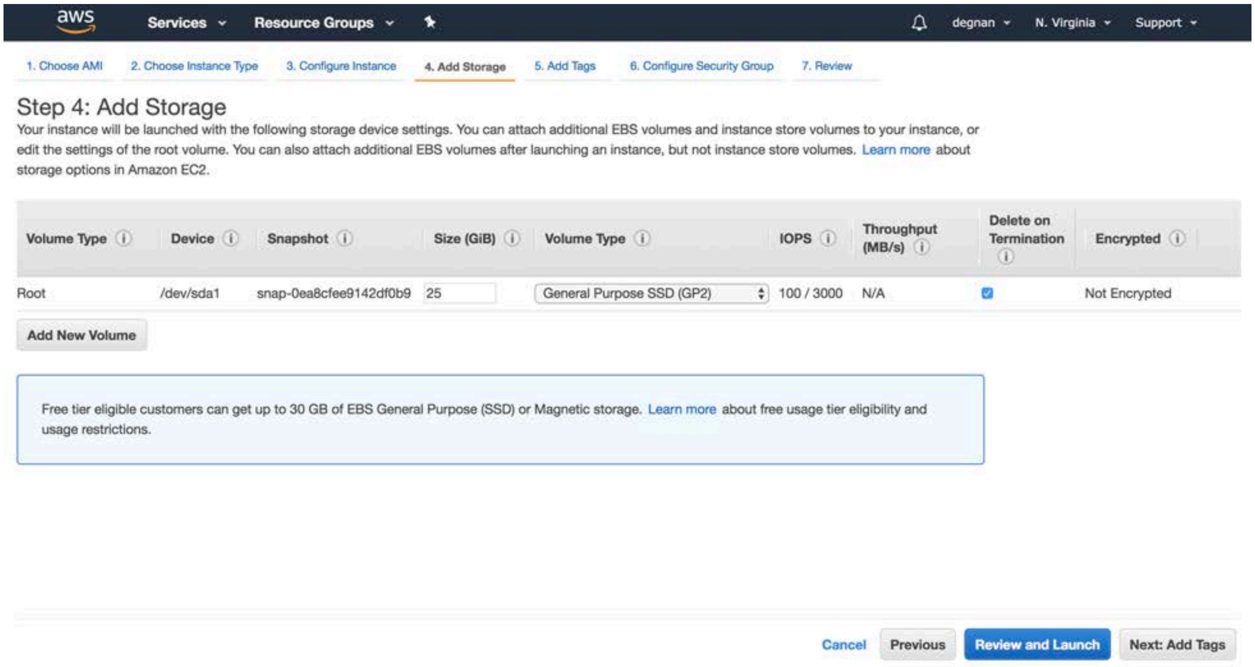


11. Select the “Next: Add Tags” button on bottom-right, to move to the next step

12. On the “Step 5: Add Tags” *optionally* hit the “Add Tag” button OR skip to step 14

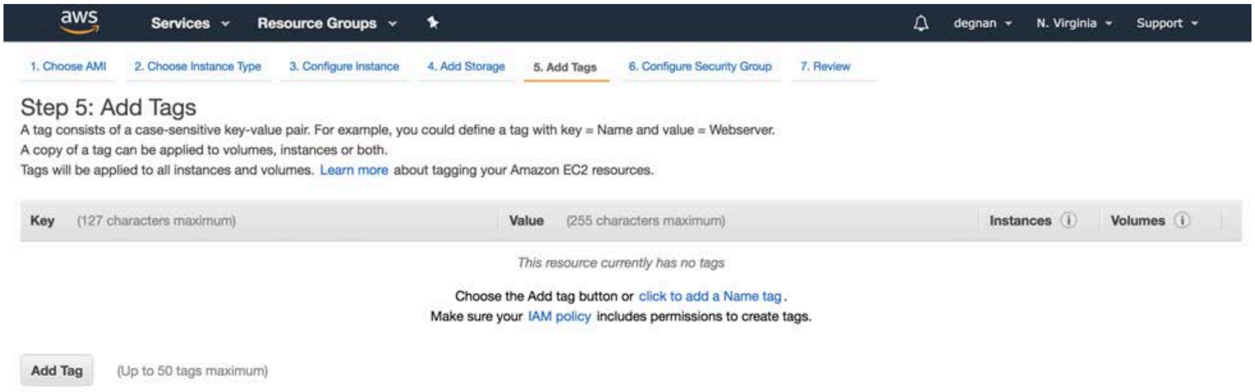


13. For example Add a *key* = “Name” and *value* = “my-SPOT” or “SPOT-server”

14. Select the “Next: Configure Security Group” button on bottom-right, to move to the next step

15. On “Step 6: Configure Security Group” page – ***leave defaults as-is***

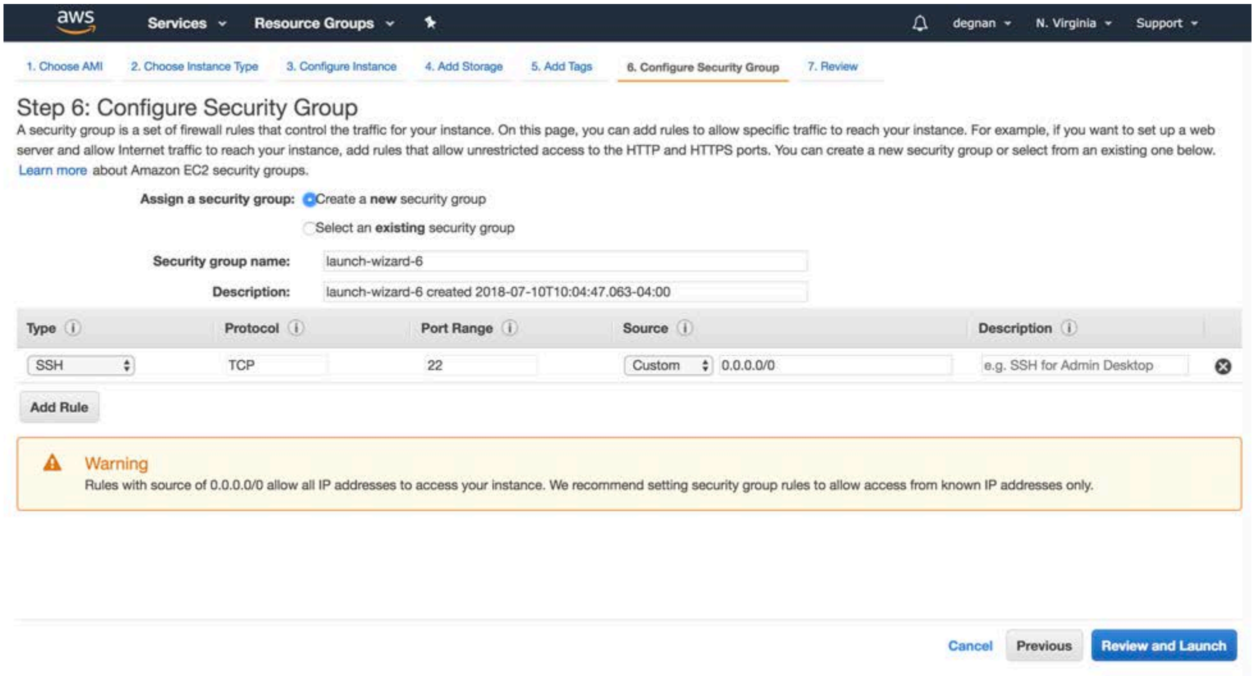


**Note: For now, we will ignore the *Warning*. In the future consider making your instances harder to access by non-users in your lab/group**

16. Select the “Review and Launch” button on bottom-right, to move to the next step

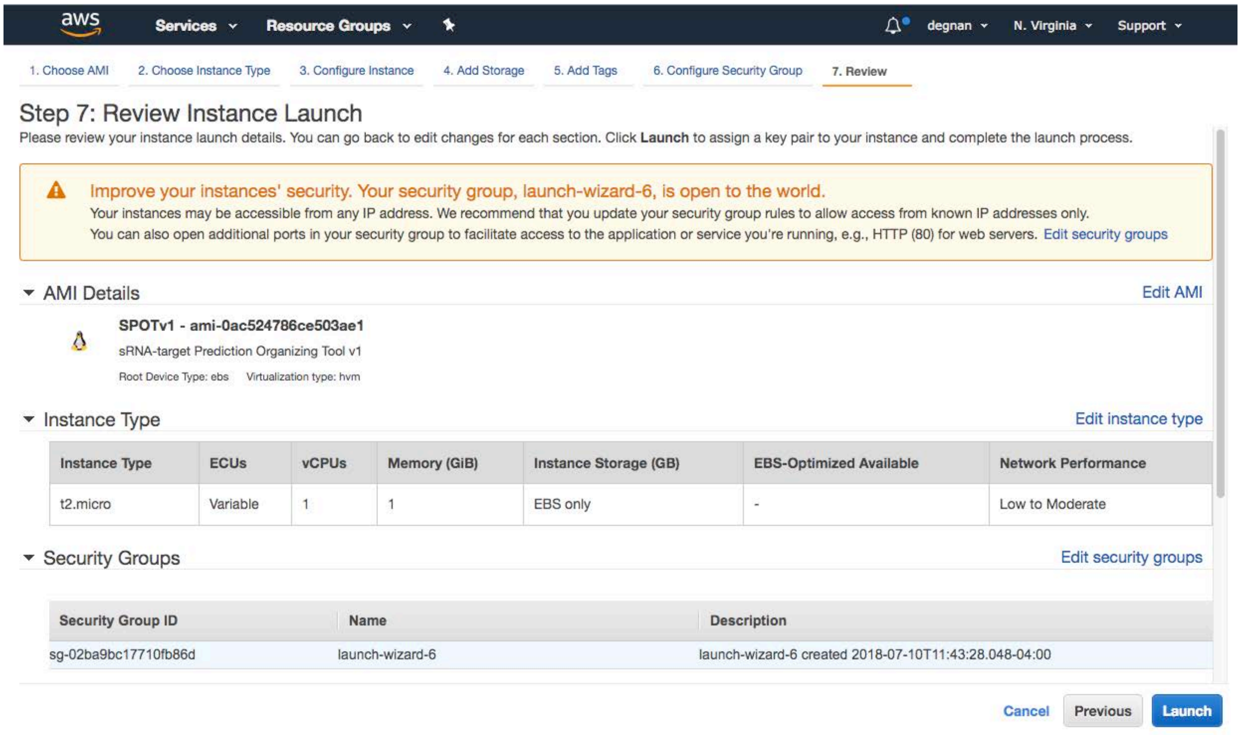

17. You can inspect the settings before hitting the "**Launch**" button. As before ignore warnings.

18. Now it asks you to select or create a key pair.

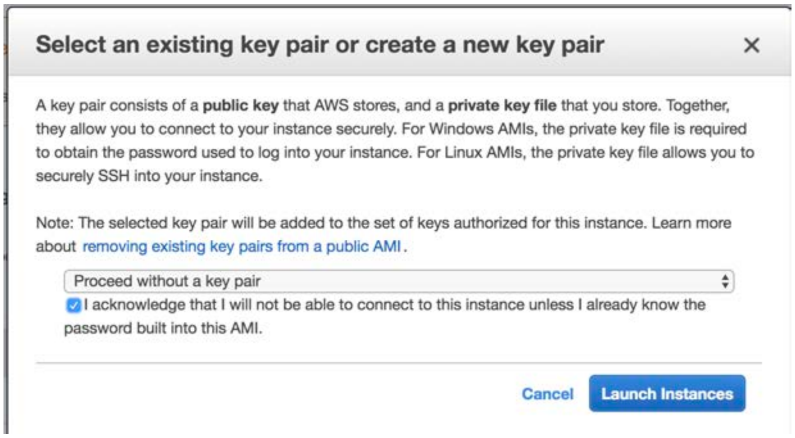


19. You will need to download the key and save it to a private location on your computer (e.g., the folder ~/.ssh/) .

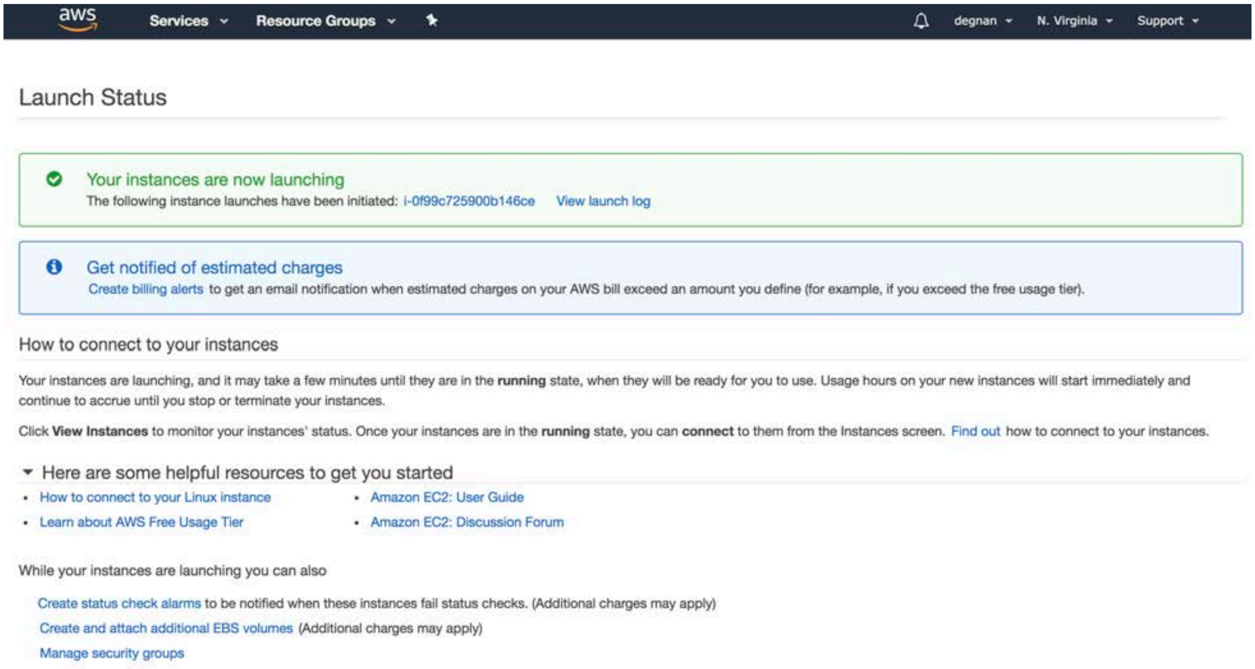


20. From here you can navigate using the left-hand side bar to your "**Instances**"

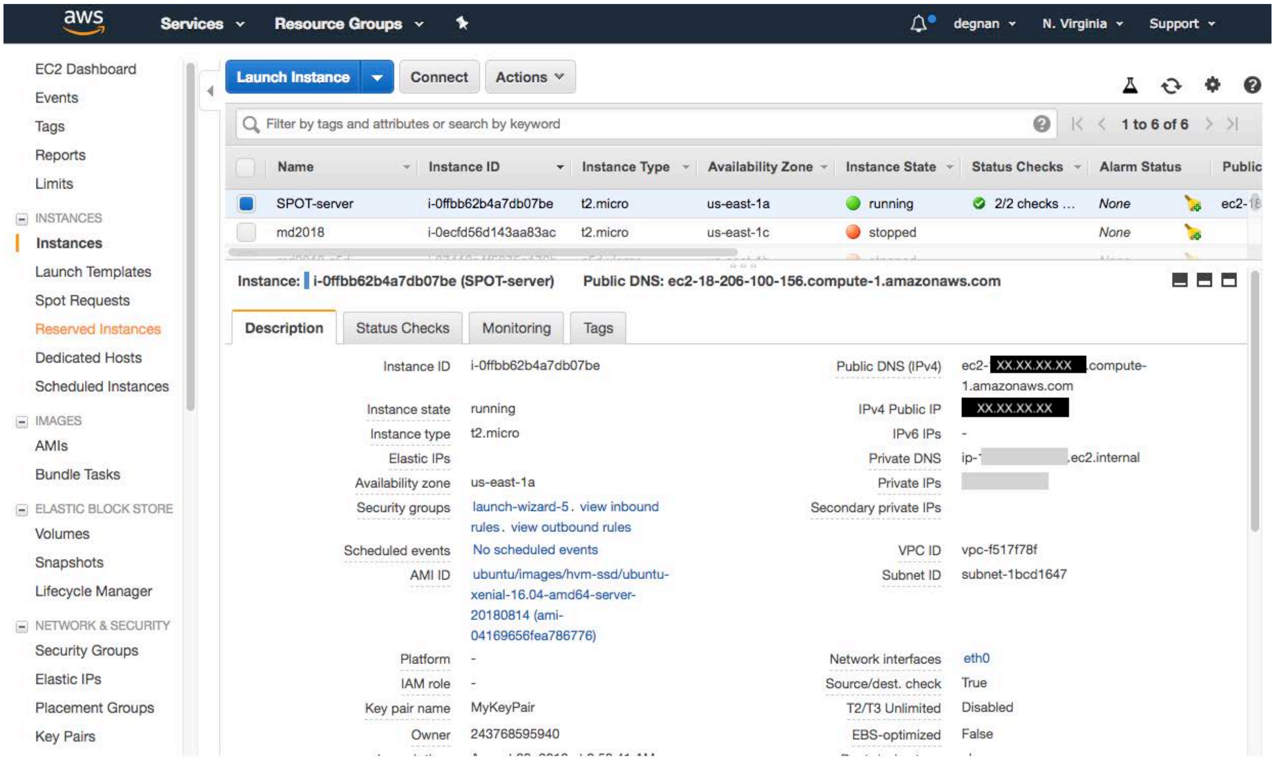


21. Instance state will be "*Initializing"* until the computer has "booted" up.

22. Once the Instance state switches to "*running*" and you select the instance, details of the instance will be shown below.

23. Find and copy the "IPv4 Public IP" address for your instance. You will use this to login to your server.

| | |
|---|---|
| IPv4 Public IP: | |

## 10. Logging into you AWS instance

To log into the server you will need your:

1. Private ssh key yourid_key.pem
2. username = first name and last initial as one word (e.g., Jane Doe = janed)
3. XX-XX-XX-XX = Your specific IPv4 Public IP from above

Login using Terminal on a **MAC or UNIX.**

$ ssh -Y -i ~/.ssh/yourid_key.pem username@XX-XX-XX-XX

Login from **Windows** using PuTTY

a. Open PuTTY
b. Under Category, click on SSH > Auth
c. Click browse

d. Find your private key (youridkey.pem) and select it
e. Under Category, click Session and input address of your EC2 instance (XX-XX-XX-XX) in the "host name" box
f. Type "SPOT" in the box under saved sessions and click save.
g. Double-click on the "SPOT" that appears under saved sessions.
h. Log in with your username. Your key should be used automatically.
i. For future logins, just double-click the "SPOT" saved session.

Once entered you will find yourself on the command line interface:

```
delta7:Desktop degnan$ ssh -Y -i ~/.ssh/MyKeyPair.pem ubuntu@18.206.100.156
Warning: No xauth data; using fake authentication data for X11 forwarding.
Welcome to Ubuntu 16.04.5 LTS (GNU/Linux 4.4.0-1065-aws x86_64)

 * Documentation:  https://help.ubuntu.com
 * Management:     https://landscape.canonical.com
 * Support:        https://ubuntu.com/advantage

  Get cloud support with Ubuntu Advantage Cloud Guest:
    http://www.ubuntu.com/business/services/cloud

2 packages can be updated.
0 updates are security updates.

New release '18.04.1 LTS' available.
Run 'do-release-upgrade' to upgrade to it.


Last login: Thu Aug 23 21:50:22 2018 from 138.23.161.215
ubuntu@ip-172-31-38-185:~$
```

## 11. References

Busch A, Richter AS, Backofen R. 2008. IntaRNA: efficient prediction of bacterial sRNA targets incorporating target site accessibility and seed regions. Bioinformatics 24:2849-2856.

Kery MB, Feldman M, Livny J, Tjaden B. 2014. TargetRNA2: identifying targets of small regulatory RNAs in Bacteria. Nucleic Acids Research 42:W124-129.

King AM, Vanderpool CK, and Degnan PH. sRNA-target Prediction Organizing Tool (SPOT) integrates computational and experimental data to facilitate functional characterization of bacterial small RNAs.

Mao X, Ma Q, Zhou C, Chen X, Zhang H,Yang J, Mao F, Lai W and Xu Y. 2013. DOOR 2.0: DOOR 2.0: presenting operons and their functions through dynamic and integrated views. Nucleic Acids Research 42: D654–D659.

McClure R, Balasubramanian D, Sun Y, Bobrovskyy M, Sumby P, Genco CA, Vanderpool CK, Tjaden B. 2013. Computational analysis of bacterial RNA-Seq data. Nucleic Acids Research 41:e140.

Wright PR, Georg J, Martin M, Sorescu DA, Richter, AS, Lott S, Kleinkauf R, Hess WR, Backofen R. 2014. CopraRNA and IntaRNA: predicting small RNA targets, networks and interaction domains. Nucleic Acids Research 42:W119-W123.

Ying X, Cao Y, Wu J, Liu Q, Cha L, Li W. 2011. sTarPicker: a method for efficient prediction of bacterial sRNA targets based on a two-step model for hybridization. PloS One 6:e22705.
