# Supplementary material for: sRNA Target Prediction Organizing Tool (SPOT) Integrates Computational and Experimental Data To Facilitate Functional Characterization of Bacterial Small RNAs
Source: mSphere. 2019 Jan 30;4(1):e00561-18. doi: 10.1128/mSphere.00561-18 (PMC6354806; doi:10.1128/mSphere.00561-18)
Supplement: TABLE S1 [file mSphere.00561-18-st001.pdf]

Table S1. Overview of confirmed and putative sRNA targets

					Primary site[a]				"Core"/Site2				
sRNA	Gene	Locus	Support	Activity	sRNA start	sRNA stop	mRNA start	mRNA stop	sRNA start	sRNA stop	mRNA start	mRNA stop	Reference
RyhB	*acnB*	b0118	interaction	repress	28	56	-19	10					Benjamin and Masse 2014
RyhB	*erpA*	b0156	interaction	repress	36	60	-12	14					Wright et al. 2013
RyhB	*fur*	b0683	interaction	repress	37	75	-96	-47					Verecrack et al. 2007
RyhB	*sdhC*	b0721	interaction	repress	43	51	-15	-7					Desnoyers and Massee 2014
RyhB	*sdhD*	b0722	predicted	repress	9	50	-42	-3					Masse and Gottesman 2002
RyhB	*marA*	b1531	interaction	repress	38	57	-11	5					Wright et al. 2013
RyhB	*fumA*	b1612	interaction	repress	38	49	-11	5					Prevost et al. 2013
RyhB	*sodB*	b1656	interaction	repress	38	46	-4	5					Afonyushkin et al. 2005
RyhB	*msrB*	b1778	interaction	repress	43	54	-35	-24	40	51	-10	3	Bos et al. 2013
RyhB	*shiA*	b1981	interaction	activate	19	75	-83	-33	44	55	-65	-54	Prevost et al. 2007
RyhB	*cirA*	b2155	interaction	activate	41	57	-58	-41					Salvail et al. 2013
RyhB	*iscS*	b2530	interaction	repress	43	69	-1	-26					Desnoyers et al. 2009
RyhB	*nirB*	b3365	interaction	repress	38	57	-9	12					Wright et al. 2013
RyhB	*cysE*	b3607	interaction	repress	34	46	-4	9					Salvail et al. 2010
RyhB	*zapB*	b3928	interaction, translational fusions	repress	51	61	24	33					Waters et al. 2017
RyhB	*frdA*	b4154	interaction, translational fusions	repress	34	47	-10	4					Waters et al. 2017
RyhB	*hdeA*	b3510	interaction, translational fusions	repress	38	50	51	62					Waters et al. 2017
RyhB	*fepB*	b0592	interaction, translational fusions	repress	45	65	-19	5					Wang et al. 2015
RyhB	*sdhA*	b0723	indirect, RyhB-sdhDC	true positive									Masse and Gottesman 2002
RyhB	*sdhB*	b0724	indirect, RyhB-sdhDC	repress									Masse and Gottesman 2002
RyhB	*iscA*	b2528	indirect, RyhB-IscS	repress									Desnoyers et al. 2009
RyhB	*iscU*	b2529	indirect, RyhB-IscS	repress									Desnoyers et al. 2009
RyhB	*iscR*	b2531	indirect, RyhB-IscS	repress									Desnoyers et al. 2009
RyhB	*carA*	b0032	no interaction	repress									
RyhB	*cra*	b0080	no interaction, co-purifies	activate									
RyhB	*uup*	b0949	no interaction	repress									
RyhB	*rpsO*	b3165	no interaction, co-purifies	activate									
RyhB	*metH*	b4019	no interaction	repress									
RyhB	*kdpA*	b0698	no interaction	repress									
RyhB	*trxA*	b3781	no interaction, co-purifies	repress									

RyhB	*yrfF*	b3398	no interaction	repress									
RyhB	*nmpC*	b0553	no interaction, co-purifies	repress									
RyhB	*ppa*	b4226	no interaction, co-purifies	repress									
Spot42	*gltA*	b0720	interaction	repress	4	13	-131	-122					Biesel and Storz 2011
Spot42	*sdhC*	b0721	interaction	repress	4	15	-56	-44					Desnoyers and Masse 2012
Spot42	*sucC*	b0728	interaction	repress	23	34	-20	-9					Wright et al. 2013
Spot42	*galK*	b0757	interaction	repress	1	61	-20	56					Moller et al. 2002
Spot42	*puuE*	b1302	interaction	repress	29	57	-35	-2					Biesel et al. 2012
Spot42	*gdhA*	b1761	interaction	repress	40	56	80	94					Wright et al. 2013
Spot42	*srlA*	b2702	interaction	repress	20	34	-15	-1					Biesel and Storz 2011
Spot42	*ascF*	b2715	interaction	repress	1	8	-28	-21	52	60	-7	2	Biesel et al. 2012
Spot42	*fucP*	b2801	interaction	repress	46	56	-39	-29					Biesel et al. 2012
Spot42	*fucI*	b2802	interaction	repress	3	57	-23	34					Biesel and Storz 2011
Spot42	*nanT*	b3224	interaction	repress	1	11	-60	-50					Biesel et al. 2012[b]
Spot42	*xylF*	b3566	interaction	repress	1	33	2	40					Biesel and Storz 2011
Spot42	*sthA*	b3962	interaction	repress	48	55	15	22					Biesel and Storz 2011
Spot42	*nanC*	b4311	interaction	repress	1	17	-24	-7					Biesel and Storz 2011
SgrS	*pstG*	b1101	interaction	repress	168	187	-30	-9					2004
SgrS	*manX*	b1817	interaction	repress	160	173	23	36					Rice et al. 2012
SgrS	*manY*	b1818	interaction	repress	168	179	-44	-30					Rice et al. 2012
SgrS	*yigL*	b3826	interaction	activate	168	187	-96	-76					Sun and Vanderpool 2013
SgrS	*asd*	b3433	interaction	repress	158	176	-31	-13	160	178	49	67	2016
SgrS	*adiY*	b4116	interaction	repress	165	179	-16	-2					2016
SgrS	*folE*	b2153	interaction	repress	158	174	-25	-12					2016
SgrS	*purR*	b1658	interaction	repress	160	179	49	67					2016
SgrS	*cusF*	b0573	no interaction, expression	repress									2016
SgrS	*nepI*	b3662	no interaction										2016
SgrS	*pliG*	b1178	no interaction, expression	activate									2016
SgrS	*bglA*	b2901	no interaction										2016
SgrS	*znuC*	b1858	no interaction, expression	activate									2016
SgrS	*cheY*	b1882	no interaction										2016
SgrS	*yeeD*	b2012	no interaction, expression	repress									2016
SgrS	*ydjN*	b1729	no interaction, expression	repress									2016
SgrS	*mglB*	b2150	no interaction, expression	activate									2016
SgrS	*mipA*	b1782	no interaction										2016
SgrS	*pstS*	b3728	no interaction										2016

SgrS	*rbsK*	b3752	no interaction										2016
RybB	*tsx*	b0411	interaction	repress	1	16	-27	-7					Gogol et al. 2011
RybB	*nmpC*	b0553	interaction	repress	1	23	-9	20					Gogol et al. 2011
RybB	*sdhC*	b0721	interaction	repress	3	15	-24	-13					Desnoyers and Masse 2012
RybB	*fiu*	b0805	interaction	repress	1	14	-84	-70					Gogol et al. 2011
RybB	*ompF*	b0929	interaction	repress	1	22	-50	-39					Gogol et al. 2011
RybB	*ompA*	b0957	interaction	repress	1	13	21	32					Gogol et al. 2011
RybB	*ompC*	b2215	interaction	repress	1	24	-63	-42					Johansen et al. 2006
RybB	*rluD*	b2594	interaction	repress	1	16	-15	5					Gogol et al. 2011
FnrS	*gpmA*	b0755	interaction	repress	38	57	-13	4					Durand and Storz 2010
FnrS	*maeA*	b1479	interaction	repress	31	65	-21	10					Durand and Storz 2010
FnrS	*marA*	b1531	interaction	repress	1	62	-18	39					Wright et al. 2013
FnrS	*sodB*	b1656	interaction	repress	1	81	-38	20					Durand and Storz 2010
FnrS	*folE*	b2153	interaction	repress	1	12	-21	-9					Durand and Storz 2010
FnrS	*folX*	b2303	interaction	repress	1	53	-24	-2					Durand and Storz 2010
FnrS	*iscR*	b2531	interaction	repress	2	54	-10	64					Wright et al. 2013
GcvB	*lrp*	b0889	interaction	repress	65	88	-21	3					Modi et al. 2011
GcvB	*phoP*	b1130	interaction	repress	148	174	-28	-7					Coornaert et al. 2013
GcvB	*oppA*	b1243	interaction	repress	60	100	-19	21					Pulvermacher et al. 2008
GcvB	*sstT*	b3089	interaction	repress	64	99	-34	2					Pulvermacher et al. 2009A
GcvB	*dppA*	b3544	interaction	repress	60	94	-42	-9					Pulvermacher et al. 2008
GcvB	*cycA*	b4208	interaction	repress	124	161	-26	7					Pulvermacher et al. 2009B
OmrA/B	*ompT*	b0565	interaction	repress	1	33	-15	20					Guillier and Gottesman 2008
OmrA/B	*csgD*	b1040	interaction	repress	2	19	-79	-61					Holmqvist E et al. (2010)
OmrA/B	*flhD*	b1892	interaction	repress	1	50	-54	-8					De Lay N, Gottesman S (2012)
OmrA/B	*cirA*	b2155	interaction	repress	2	24	-35	-10					Guillier and Gottesman 2008
OmrA/B	*ompR*	b3405	interaction	repress	1	18	-29	-11					Guillier and Gottesman 2008
CyaR	*ompX*	b0814	interaction	repress	38	48	-9	2					De Lay and Gottesman 2009
CyaR	*nadE*	b1740	interaction	repress	39	49	-11	-2					De Lay and Gottesman 2009
CyaR	*yobF*	b1824	interaction	repress	1	43	-25	19					Wright et al. 2013
CyaR	*yqaE*	b2666	interaction	repress	31	43	4	16					De Lay and Gottesman 2009
CyaR	*luxS*	b2687	interaction	repress	40	49	-12	-3					De Lay and Gottesman 2009
DicF	*ftsZ*	b0095	interaction	repress	25	52	-28	-1					Balasubramanian et al. 2016
DicF	*pykA*	b1854	interaction	repress	25	43	-19	1					Balasubramanian et al. 2016
DicF	*xylR*	b3569	interaction	repress	6	14	10	19					Balasubramanian et al. 2016
DicF	*pfkA*	b3916	interaction	repress	34	47	-11	2					Balasubramanian et al. 2016

MicA	*ompA*	b0957	interaction	down	8	24	-21	-6					Gogol et al. 2011
MicA	*ompX*	b0814	interaction	down	1	22	1	23					Gogol et al. 2011
MicA	*phoP*	b1130	interaction	down	6	31	-15	8					Coornaert et al. 2010
MicA	*tsx*	b0411	interaction	down	1	21	-58	-36					Gogol et al. 2011
MicF	*phoE*	b0241	interaction	down	2	14	21	33					Holmqvist et al. 2012
MicF	*lrp*	b0889	interaction	down	1	13	-2	12					Holmqvist et al. 2012
MicF	*ompF*	b0929	interaction	down	1	33	-16	10					Urban et al. 2007, Anderson et al. 1990, Schmidt et al. 1995,Chen et al. 2004
MicF	*cpxR*	b3912	interaction	down	5	16	18	29					Holmqvist et al. 2012
RydC	*cfa*	b1661	interaction	activate	2	12	-109	-99					Frohlich et al. 2013
RydC	*csgD*	b1040	interaction	repress	8	20	13	1					Bordeau and Felden 2014

[a] Coordinates sRNA 5'->3', mRNA based on AUG

[b] Author's coordinates based on upstream GUG start codon instead of AUG
