# Supplementary material for: sRNA Target Prediction Organizing Tool (SPOT) Integrates Computational and Experimental Data To Facilitate Functional Characterization of Bacterial Small RNAs
Source: mSphere. 2019 Jan 30;4(1):e00561-18. doi: 10.1128/mSphere.00561-18 (PMC6354806; doi:10.1128/mSphere.00561-18)
Supplement: TABLE S2 [file mSphere.00561-18-st002.pdf]

Table S2. *E. coli* MG1655 known small RNAs

Gene	Locus ID[a]	EcoGene No.	Start	Stop	Strand	Length (nt)	RFAM	Product/Description
sokC	b4413	EG31128	16901	17053	-	153	RF01794	sok antitoxin
sgrS	b4577	EG31149	77367	77593	+	227	RF00534	sRNA antisense regulator destabilzes ptsG mRNA, Hfq-dependent; regulated by sgrR
tff	b4414	EG31135	189712	189847	+	136	RF00127	novel sRNA, function unknown; identified in a large scale screen
eyeA	b4690	EG31183	272580	272654	+	75		novel sRNA, function unknown, CP4-6 propahge
ffs	b0455	EG30027	476448	476561	+	114	RF00169	4.5S sRNA component of Signal Recognition Particle (SRP); 4.5S RNA; component of ribonucleoprotein particle; works with the Ffh protein; adjusted endpoints to reflect the mature 4.5S RNA (114 nt)
chiX, sroB	b4585	EG31175	507204	507287	+	84	RF00368	sRNA antisense regulator of OM chitoporin ChiP(YbfM), Hfq-dependent
iepX			574365	574531	-	167		inhibitor of porin expression (Castillo-Keller et al. 2006)
sokE	b4700	EG31186	607689	607838	+	150	RF01794	sok antitoxin
sroC	G0-9383		686681	686843	-	163	RF00369	sroC RNA
rybA	b4416		852952	853040	-	89		small RNA RybA
rybB	b4417	EG31137	887976	888054	-	79	RF00110	sRNA antisense regulator of ompC and ompW mRNA instability, Hfq-dependent; identified in a large scale screen
psrD	b4418	EG31138	1146589	1146757	+	169	RF00077	novel Novel sRNA, function unknown; identified in a large scale screen
rdlA	b4420	EG31139	1269323	1269389	+	67	RF01813	sRNA antisense regulator affects LdrA translation; proposed addiction module in LDR-A repeat, with toxic peptide LdrA; antisense RNA, trans-acting regulator of ldrA translation
rdlB	b4422	EG31140	1269858	1269923	+	66	RF01813	sRNA antisense regulator affects LdrB translation; proposed addiction module in LDR-B repeat, with toxic peptide LdrB; antisense RNA, trans-acting regulator of ldrB translation
rdlC	b4424	EG31141	1270393	1270460	+	68	RF01813	sRNA antisense regulator affects LdrC translation; proposed addiction module in LDR-C repeat, with toxic peptide LdrC; antisense RNA, trans-acting regulator of ldrC translation
rttR	b4425	EG31131	1287066	1287236	-	171	RF00391	rtT sRNA, processed from tyrT transcript; rtT RNA may modulate the stringent response; released from primary tyrT transcript during tRNA processing; encodes putative Tpr protein
RNA0-359	G0-10697		1299474	1300927	+	255		small RNA

mcaS	b4426	EG31143	1405656	1405751	-	96	RF00115	motility and biofilm regulator sRNA; identified in a large scale screen
fnrS, ECS161	b4699	EG31185	1409129	1409250	+	122	RF01796	FNR-activated anaerobic sRNA antisense regulator down regulates cydDC, metE, sodA, sodB, Hfq-dependent; mediates negative FNR regulation
ralA	b4714	EG31192	1413556	1413734	+	179		antitoxin RalA
micC	b4427	EG31144	1437118	1437238	+	121	RF00121	MicC RNA
rydC	b4597	EG31168	1491443	1491506	-	64	RF00505	sRNA antisense regulator of csgD and yejABEF, Hfq-dependent
sokB	b4429	EG31127	1492066	1492217	-	152	RF01794	sok antitoxin
mgrR	b4698	EG31184	1622735	1622948	-	214	RF01407	STnc560 Hfq binding RNA
dicF	b1574	EG31115	1649382	1649434	+	53	RF00039	sRNA antisense regulator, inhibits ftsZ, Hfq-dependent; Qin prophage; DicF antisense RNA, inhibits ftsZ translation
RNA0-360	G0-10698		1670949	1671136	-	188		small RNA
rydB	b4430	EG31145	1764713	1764780	-	68	RF00118	novel sRNA, function unknown; identified in a large scale screen
rprA	b4431	EG31130	1770372	1770477	+	106	RF00034	sRNA antisense activator for RpoS translation, Hfq-dependent; RprA RNA; positive regulatory RNA for rpoS translation
sroD	G0-9384		1888017	1888102	-	86	RF00370	sroD RNA
ryeA	b4432	EG31146	1923066	1923314	+	249	RF00101	novel sRNA, function unknown; identified in a large scale screen
sdsR, ryeB	b4433	EG31147	1923164	1923284	-	121	RF00111	novel sRNA, function unknown; identified in a large scale screen
micL-S	b4717		1958441	1958748	-	308		small regulatory RNA MicL-S
3'ETSleuZ	G0-16636		1991748	1991817	-	70		3'ETSleuZ
sdsN, RNA0-361	b4719		1996906	1997097	+	192		small regulatory RNA SdsN
dsrA	b1954	EG31118	2025227	2025313	-	87	RF00014	sRNA antisense regulator enhances translation of RpoS, Hfq-dependent; silencer of rcsA gene, interacts with rpoS translation; component of acid resistance regulatory circuit
rseX	b4603	EG31170	2033649	2033739	+	91	RF01401	sRNA antisense regulator of ompA and ompC translation, Hfq-dependent
isrC	b4435	EG31150	2071317	2071511	+	195	RF00124	novel sRNA, CP4-44; putative prophage remnant; identified in a large scale screen
sibA, ryeC	b4436	EG31151	2153309	2153451	+	143	RF00113	sRNA antisense regulator of toxic IbsA protein; identified in a large scale screen
sibB, ryeD	b4437	EG31152	2153644	2153779	+	136	RF00113	sRNA antisense regulator of toxic IbsB protein; identified in a large scale screen

cyaR	b4438	EG31153	2167114	2167200	+	87	RF00112	sRNA antisense regulator of ompX mRNA instability, Hfq-dependent, cAMP-induced; identified in a large scale screen
RNA0-362,ECS026	G0-10700		2278258	2278498	-	241		small RNA
micF	b4439	EG30063	2313084	2313176	+	93	RF00033	sRNA antisense regulator affecting ompF expression, Hfq-dependent; regulatory antisense RNA affecting ompF expression; member of soxRS regulon
ryeG, ES132	G0-16635		2470466	2470665	-	200		small regulatory RNA RyeG
ryfA	b4440	EG31154	2653855	2654158	+	304	RF00126	novel sRNA, function unknown; identified in a large scale screen
glmY	b4441	EG31155	2691157	2691340	-	184	RF00083	sRNA activator of glmS mRNA, glmZ processing antagonist; identified in a large scale screen
ohsC, ryfC	b4608	EG31178	2700520	2700596	+	77	RF02495	sRNA antisense regulator of shoB toxin
ryfD	b4609	EG31179	2734153	2734295	-	143		novel sRNA, function unknown
ssrA	b2621	EG30100	2755593	2755955	+	363	RF00023	tmRNA, 10Sa RNA; acts as tRNA-Ala and mRNA template for tagging proteins resulting from premature transcription termination for degradation, a process known as trans-translation
micA	b4442	EG31156	2814802	2814879	+	78	RF00078	sRNA antisense regulator of ompA, lamB, ompX, and phoP; Hfq-dependent; identified in a large scale screen
sokX	b4701	EG31187	2887300	2887455	-	156	RF01794	sok antitoxin
csrB	b4408	EG31119	2924156	2924524	-	369	RF00018	CsrA-binding sRNA, antagonizing CsrA regulation; CsrB regulatory RNA; binds global regulatory protein CsrA, antagonizing CsrA regulation, blocking the CsrA binding of glgC mRNA
gcvB	b4443	EG31129	2942696	2942901	+	206	RF00022	sRNA antisense regulator represses oppA, dppA, gltI and livJ expression, Hfq-dependent; regulated by gcvA and gcvR
omrA	b4444	EG31157	2976102	2976189	-	88	RF00079	sRNA antisense regulator downregulates OM proteins and curli; positively regulated by OmpR/EnvZ, Hfq-dependent; identified in a large scale screen
omrB	b4445	EG31158	2976304	2976385	-	82	RF00079	sRNA antisense regulator downregulates OM proteins and curli; positively regulated by OmpR/EnvZ, Hfq-dependent; identified in a large scale screen
ssrS, 6S	b2911	EG30099	3055983	3056165	+	183	RF00013	6S RNA inhibits RNA polymerase promoter binding; template for RNA-directed pRNA synthesis by RNAP; mimics an open promoter
sibC, rygC	b4446	EG31159	3056849	3056988	+	140	RF00113	sRNA antisense regulator of toxic IbsC protein; identified in a large scale screen
och5, RNA0-363, ECS181	G0-10701		3067187	3067344	+	158		small regulatory RNA Och5

sibD, rygD	b4447	EG31160	3194723	3194865	-	143	RF00113	sRNA antisense regulator of toxic IbsD protein; identified in a large scale screen
sibE, rygE	b4611	EG31169	3195099	3195240	-	142	RF00113	sRNA antisense regulator of toxic IbsE protein
rnpB	b3123	EG30069	3270216	3270592	-	377	RF00010	RNase P, M1 RNA component; RNase P is an essential enzyme responsible for the processing of the 5' ends of precursors to tRNA, 4.5S RNA, and other naturally occurring stable RNA molecules; RNase P also affects gene expression of polycistronic operon mRNAs
sraG, psrO	b4449	EG31162	3311225	3311398	+	174	RF00082	novel sRNA, function unknown; identified in a large scale screen
arcZ	b4450	EG31163	3350577	3350697	+	121	RF00081	sRNA positive antisense regulator of rpoS; binds Hfq; identified in a large scale screen
ES185, RNA0-364	G0-10702		3354062	3354227	+	166		small RNA
RNA0-365, ES186	G0-10703		3367613	3367770	-	158		small RNA
ryhB	b4451	EG31133	3580927	3581016	-	90	RF00057	sRNA antisense regulator mediating positive Fur regulon response, Hfq-dependent; global iron regulator; Fur represses this inhibitory RNA, relieving acnA, fumA, sdhCDAB, bfr, ftnA, and sodB from RyhB-mediated repression; gvcB is regulated by gcvA and gcvR
agrA	b4712	EG31189	3648063	3648146	+	84		inactive antisense sRNA
agrB, ECS004	b4713	EG31190	3648294	3648377	+	84		antisense sRNA antitoxin for the DinQ toxin
arrS	b4704	EG31188	3657986	3658054	-	69		antisense sRNA ArrS, function unknown
gadF, ES205	b4718		3658992	3659082	+	91		small regulatory RNA GadF
gadY	b4452	EG31164	3664861	3664974	+	114	RF00122	GadY
rdlD	b4454	EG31142	3700136	3700201	+	66	RF01813	rdlD antitoxin
rirA	G0-16662		3808182	3808220	-	39	RF01707	JUMPstart RNA
istR	b4616	EG31167	3853118	3853257	-	140	RF01400	sRNA antisense regulators IstR-1 and IstR-2, affectr tisB, Hfq-dependent
glmZ	b4456	EG31165	3986432	3986638	+	207	RF00083	sRNA antisense activator of glmS mRNA, Hfq-dependent; identified in a large scale screen
RNA0-366	G0-10704		4001191	4001334	+	144		small RNA
esrE	b4707	EG31191	4019978	4020229	+	252		putative sRNA of unknown function
spf	b3864	EG30098	4049899	4050007	+	109	RF00021	Spot 42 sRNA antisense regulator of galK translation, Hfq-dependent; RIP293 (repetitive extragenic palindromic) element; contains 2 REP sequences and 1 IHF site
csrC	b4457	EG31134	4051036	4051280	+	245	RF00084	CsrC sRNA sequesters CsrA, a carbon flux regulator; CsrC regulatory RNA; binds global regulatory protein CsrA, antagonizing CsrA regulation
RNA0-367	G0-10705		4058171	4058242	+	72		small RNA

cpxQ	b4716		4106330	4106387	+	58		small regulatory RNA CpxQ
oxyS	b4458	EG31116	4158285	4158394	-	110	RF00035	sRNA antisense regulator activates genes that detoxify oxidative damage, Hfq-dependent; global regulatory RNA OxyS
sroH	b4691	EG31182	4190327	4190487	-	161	RF00372	novel sRNA, function unknown
ryjA, sraL	b4459	EG31166	4277927	4278066	-	140	RF01408	novel sRNA, function unknown; identified in a large scale screen
ryjB	b4624	EG31181	4527977	4528066	+	90		novel sRNA, function unknown
symR	b4625	EG31180	4579835	4579911	+	77	RF01809	sRNA antisense regulator destabilizing divergent and overlapping symE mRNA

[a] Ecocyc.org specific entries have Locus IDs starting with 'GO'
