# Supplementary material for: sRNA Target Prediction Organizing Tool (SPOT) Integrates Computational and Experimental Data To Facilitate Functional Characterization of Bacterial Small RNAs
Source: mSphere. 2019 Jan 30;4(1):e00561-18. doi: 10.1128/mSphere.00561-18 (PMC6354806; doi:10.1128/mSphere.00561-18)
Supplement: TABLE S3 [file mSphere.00561-18-st003.pdf]

Table S3. Strains and plasmids used in this study

**Strain**	**Description**	**Source**
MG1655	Wild-type E. coli K-12	D. Jin, NCI
DJ480	MG1655 *Δlac* X74	D. Jin, NCI
JH111	*ΔsgrS*, λattB::lacIq, tetR(ep), spR	(37)
PM1205	lacI::PBAD:cat-sacB:lacZ, *ΔaraBAD*, araC+, mal::lacIq, miniλ tet; grow at 30°C	(29)
AK250	*ΔrydC::kan,* λattB::lacIq	This study
AK27	pBAD-212UTR-*cfa'-'lacZ*	This study
AK28	pBAD-34UTR-*cfa'-'lacZ*	This study
AK42	pBAD-52UTR-*yejA'-'lacZ*	This study
AK66	Hfq3XFLAG, rne131::kan	This study
AK99	pBAD-148UTR-*csgD'-'lacZ*	This study
AK106	pBAD-100UTR-*pheA*(mut-67)*'-'lacZ*	This study
AK109	pBAD-100UTR-*pheA'-'lacZ*	This study
AK112	pBAD-110UTR-*lldP'-'lacZ*	This study
AK113	pBAD-30UTR-*trpE'-'lacZ*	This study
AK115	pBAD-40UTR-*yibT'-'lacZ*	This study
AK119	pBAD-79UTR-*moaB'-'lacZ*	This study
AK121	pBAD-30UTR-*trpE*(mut-20)*'-'lacZ*	This study
AK123	pBAD-62UTR-*yhjD'-'lacZ*	This study
AK124	pBAD-20UTR-*araH'-'lacZ*	This study
AK125	pBAD-43UTR-*purK'-'lacZ*	This study
AK127	pBAD-74UTR-*cysQ'-'lacZ*	This study
AK128	pBAD-39UTR-*grpE'-'lacZ*	This study
AK129	pBAD-137UTR-*ldtD'-'lacZ*	This study
AK130	pBAD-139UTR-*lldR'-'lacZ*	This study
AK131	pBAD-30UTR-*trunc_trpE'-'lacZ*	This study
AK132	pBAD-28UTR-*ygaU'-'lacZ*	This study
AK133	pBAD-30UTR-*moaC'-'lacZ*	This study
**Plasmid**		
pBRCS12	pHDB3, P_{LlacO}, vector control	Wadler and Vanderpool 2009
pLAK1	pHDB3, P_{LlacO}-*rydC*	This study
pLAK2	pHDB3, P_{LlacO}-*rydC3*	This study
pLAK3	pHDB3, P_{LlacO}-*rydC5*	This study
pLAK4	pHDB3, P_{LlacO}-*rydC345*	This study
pLAK5	pHDB3, P_{LlacO}-*rydC-MS2*	This study
