# Supplementary material for: sRNA Target Prediction Organizing Tool (SPOT) Integrates Computational and Experimental Data To Facilitate Functional Characterization of Bacterial Small RNAs
Source: mSphere. 2019 Jan 30;4(1):e00561-18. doi: 10.1128/mSphere.00561-18 (PMC6354806; doi:10.1128/mSphere.00561-18)
Supplement: TABLE S4 [file mSphere.00561-18-st004.pdf]

Table S4. Oligos used in this study

Oligo	Sequence 5′ - 3′
O-AK24F	ACCTGACGCTTTTTATCGCAACTCTCTACTGTTTCTCCATGAGTTGTTAAAG CTACGATAAATATTATGTTTTTACGGGGACAGGATCGT
O-AK24F2	ACCTGACGCTTTTTATCGCAACTCTCTACTGTTTCTCCATAAGGTTCTGATC ACCGACCAGTGATGGAGAAA
O-AK24R	TAACGCCAGGGTTTTCCCAGTCACGACGTTGTAAAACGACCGGGGCAGAA CCGTTAATGGCTATACC
O-AKMF	GATCGATCGGATCCCTTCCGATGTAGACCCGTAT
O-AKMR	GATCGATCAAGCTTCAGACCCTGATGGTGTCTGAAAAACGTACCCTGATG GTGTACGAAGAAAACGCCTGTACTAAAACCGA
O-AK31F	ACCTGACGCTTTTTATCGCAACTCTCTACTGTTTCTCCATTGAAAGCATGCC GGATTGCG
O-AK31R	TAACGCCAGGGTTTTCCCAGTCACGACGTTGTAAAACGACTGCAATGGCTT TGACGGTGG
O-AK37F	ACCTGACGCTTTTTATCGCAACTCTCTACTGTTTCTCCATCAGATGTAATCC ATTAGTTTTATATTTTACCCATTTAGGG
O-AK37R	TAACGCCAGGGTTTTCCCAGTCACGACGTTGTAAAACGACAAGGTGCTGC AAGAGAGCTG
O-AK59F	TCTTCGCCTGTACCACCGCTCGGTTTTAGTACAGG
O-AK59R	CCTGTACTAAAACCGAGCGGTGGTACAGGCGAAGA
O-AK67F	ACCTGACGCTTTTTATCGCAACTCTCTACTGTTTCTCCATGAATGGGAGGC GTTTCGTCG
O-AK67Mut	ACCTGACGCTTTTTATCGCAACTCTCTACTGTTTCTCCATGGCAACACTATG ACATCCCAAAACCCG
O-AK67R	TAACGCCAGGGTTTTCCCAGTCACGACGTTGTAAAACGACGCATTGCTGG AATGGCTGCG
O-AK68F	ACAAGATACTGACGTCCTTGGGATGTAGACCCGTATTCTT
O-AK68R	AAGAATACGGGTCTACATCCCAAGGACGTCAGTATCTTGT
O-AK69F	ATAACAAGATACTGACGTCCTAGGGATGTAGACCCGTATTCTTCG
O-AK69R	CGAAGAATACGGGTCTACATCCCTAGGACGTCAGTATCTTGTTAT
O-AK78F	ACCTGACGCTTTTTATCGCAACTCTCTACTGTTTCTCCATGTCATTATCCCT ACACAACACAATTGGC
O-AK78R	TAACGCCAGGGTTTTCCCAGTCACGACGTTGTAAAACGACGGGCCACAAC CCGTAGAA
O-AK80	TCGCAACTCTCTACTGTTTCTCCATCTTTTTTTTGAACAAAATTAGAGAATAA CAATGCAAACACAAAAACCGACTCTCGAACTGCTAACCTGCGAAGGCGTC GTTTTACAACGTCGTGACTGGG
O-AK80Mut	TCGCAACTCTCTACTGTTTCTCCATCTTTTTTTTGAACAAAATTAGAGAATAA CAATGCAAACACAAAAACCGAGTGTCGAACTGCTAACCTGCGAAGGCGTC GTTTTACAACGTCGTGACTGGG
O-AK80tru	TCGCAACTCTCTACTGTTTCTCCATCTTTTTTTTGAACAAAATTAGAGAATAA CAATGGTCGTTTTACAACGTCGTGACTGGG
O-AK81	TCGCAACTCTCTACTGTTTCTCCATGCCGCGCATTCCCGGCGCGGGGTAA TACGGAGATATCATCATGGGCAAATTAGGTGAAGTCGTTTTACAACGTCGT GACTGGG
O-AK82	TCGCAACTCTCTACTGTTTCTCCATTTCCTGCATCAAAACAACACCGGTATT ACGCAAAACTTATCGTACATTGGCGGCTAAAACGTCAAAAGGAGAGATCA GATGAGTCAGGTAAGCACTGAATTTATCCCGACCCGTATTGCTATTCTTGT CGTTTTACAACGTCGTGACTGGG
O-AK83	TCGCAACTCTCTACTGTTTCTCCATAGTACCTGGACTGACCGACTATACTTT TTAAGAATGACCACGACAGAAGGACAAAAGAGCGGATGACGGTCGTTTTA CAACGTCGTGA CTGGG

O-AK84	TCGCAACTCTCTACTGTTTCTCCATGCCTGAGTAAGGAGAGTATGATGTCT<br>TCTGTTTCTACATCGGGGTCTGGCGTCGTTTTACAACGTCGTGACTGGG
O-AK85	TCGCAACTCTCTACTGTTTCTCCATAGACCGACGAAGTGCTGGAAAACCCG<br>GACCCGCGAGGTGCGGCATGAAACAGGTTTGCGTCCTCGGTGTCGTTTTA<br>CAACGTCGTGACTGGG
O-AK86	TCGCAACTCTCTACTGTTTCTCCATGTTGGCGCATTCATTAACGATAGGGT<br>ATAAGTAAAACAATAAGTTAACACCGCTCACAGAGACGAGGTGGAGAAATG<br>TTAGATCAAGTATGCCAGCTTGCACGGAATGCAGGCGATGCCATTATGCA<br>GGTCTACGACGGGACGGTCGTTTTACAACGTCGTGACTGGG
O-AK87	TCGCAACTCTCTACTGTTTCTCCATAGTTAGCGAGATGAATGCGAAAAAAA<br>CGCGGAGAAATTCATGAGTAGTAAAGAACAGAAAACGCCTGAGGGGCAAG<br>CCGTCGTTTTACAACGTCGTGACTGGG
O-AK88	TCGCAACTCTCTACTGTTTCTCCATCGGCGTTTATATACTGAAGATAAGCCT<br>GATGAGTAACAGGCTTGCTCGTCATACTTTCGTGAGTATTGGCGTTGTACA<br>GGCAAGTCGTAAAATAACAGCCTGGCTATTCAGAGTATGATAAAAACAGGG<br>GGCAAGGGATGTTGGTCGTTTTACAACGTCGTGACTGGG
O-AK89	TCGCAACTCTCTACTGTTTCTCCATTCGCCTGTGCGGCGGTAGGCCTGGT<br>GGGCAAAGAGTCTGATTTGTTCCGCTTTACTGTCAAACACAGCCTGATCTT<br>CACCTGTATAGTGGGCGTGATCACCACGCTTCAGGCTTATGTCTTAACGTG<br>GATGATTCCTTAATGATTGTTTTACCCAGACGCCTGTCAGACGAGGTTGCC<br>GATCGTGTGCGGGCGCTGATTGATGTCGTTTTACAACGTCGTGACTGGG
O-AK90	TCGCAACTCTCTACTGTTTCTCCATAGTGTTTCCGTTAACGAATGAGGTAG<br>CTATGGGTCTGTTCAATTTTGTGAAAGATGCCGGAGAAAAACTCTGGGACG<br>CGGTTACAGGTCAGCACGATAAAGACGATCAGGCGAAGAAGGTGCAGGA<br>GGTCGTTTTACAACGTCGTGACTGGG
O-AK91	TCGCAACTCTCTACTGTTTCTCCATTAATTTCCATCCACATTTGAAGAAATA<br>AGTATGTCGCAACTGACCCATATCGTCGTTTTACAACGTCGTGACTGGG
